# Proteomic and molecular dynamic investigations of PTM-induced structural fluctuations in breast and ovarian cancer

**DOI:** 10.1038/s41598-021-98201-7

**Published:** 2021-09-29

**Authors:** Dmitry Tikhonov, Liudmila Kulikova, Arthur T. Kopylov, Vladimir Rudnev, Alexander Stepanov, Kristina Malsagova, Alexander Izotov, Dmitry Kulikov, Alexey Zulkarnaev, Dmitry Enikeev, Natalia Potoldykova, Anna L. Kaysheva

**Affiliations:** 1 Institute of Mathematical Problems of Biology RAS-the Branch of Keldysh Institute of Applied Mathematics of Russian Academy of Sciences, 142290 Pushchino, Moscow Region Russia; 2 Institute of Theoretical and Experimental Biophysics, Russian Academy of Sciences, 142290 Pushchino, Moscow Region Russia; 3 V.N. Orekhovich Institute of Biomedical Chemistry, 119121 Moscow, Russia; 4 Moscow Regional Research and Clinical Institute, Russian Federation, 129110 Moscow, Russia; 5 Institute of Urology and Reproductive Health, Sechenov University, 119121 Moscow, Russia

**Keywords:** Breast cancer, Chemical modification, Protein folding, Molecular modelling

## Abstract

Post-translational processing leads to conformational changes in protein structure that modulate molecular functions and change the signature of metabolic transformations and immune responses. Some post-translational modifications (PTMs), such as phosphorylation and acetylation, are strongly related to oncogenic processes and malignancy. This study investigated a PTM pattern in patients with gender-specific ovarian or breast cancer. Proteomic profiling and analysis of cancer-specific PTM patterns were performed using high-resolution UPLC-MS/MS. Structural analysis, topology, and stability of PTMs associated with sex-specific cancers were analyzed using molecular dynamics modeling. We identified highly specific PTMs, of which 12 modified peptides from eight distinct proteins derived from patients with ovarian cancer and 6 peptides of three proteins favored patients from the group with breast cancer. We found that all defined PTMs were localized in the compact and stable structural motifs exposed outside the solvent environment. PTMs increase the solvent-accessible surface area of the modified moiety and its active environment. The observed conformational fluctuations are still inadequate to activate the structural degradation and enhance protein elimination/clearance; however, it is sufficient for the significant modulation of protein activity.

## Introduction

Epithelial ovarian cancer accounts for up to 90% of all malignant ovarian neoplasms. Moreover, breast cancer commonly affects milk ducts and lobules (also known as invasive ductal carcinoma). It can also originate from glandular tissue (invasive lobular carcinoma) and germ cells^1^. Breast cancer is the most prevalent type of tumor in women and is the second most common cancer type in general^2^. Determination of molecular events associated with the onset and progression is a major task due to the lack of credible markers for early diagnostic and limited success in treatment strategy. As a result, high disability and mortality levels are arising^2^.

The transformation of normal cells into the neoplastic one is accompanied by various endogenous molecular events, which are generally orchestrated by the comprehensive and dynamic network of post-translational modifications (PTM)^3^. PTMs are thought to play a pivotal role in the maintenance of different biological processes; thus, disruption of PTM crosstalk initiates a sequence of oncogenic events. Several PTM moieties can be represented on the protein surface and create a “PTM code.” The proper “code” is important for the organization of intracellular signaling through interaction with various effectors. Therefore, different strategies and proteomic tools, including affinity-based separation, TiO_2_ enrichment, and the HDX technique, have been recently developed to decipher distinct PTMs and determine their localization on the protein surface. To date, up to 450 different PTMs have been distinguished and annotated. The most prevalent PTMs are phosphorylation, acetylation, methylation, and ubiquitination^4^. Less known citrullination and SUMOylation can be favorable due to alterations in CD4^+^ T-cells in response to cell stress, immune activation^5^, sensing of DNA damage response, and telomere maintenance^6^.

The main source of PTM identification is mass spectrometry-based proteomics, supplied by integration with interactome and transcriptome approaches. However, mass spectrometry data are highly redundant, and search engines are imperfect, whereas validation of the putatively identified PTMs by immunochemistry is limited. The broad dynamic range is another significant challenge in plasma/serum proteomics, making identification of modified proteins rather limited. This issue can be overcome by immunodepleting, but this tool is poorly reproducible and requires large amount of initial material to enrich modified proteins sufficiently. Altogether, these challenges lead to several misidentifications and significantly hinders investigation of the exact role of specific PTMs in oncogenesis. Therefore, only a small fraction of PTMs is well validated and is related to several types of cancer, including glycosylation of COX-2 in colorectal cancer^7^, citrullination of fibronectin during renal cancer^8^, phosphorylation of PKM2 in thyroid cancer^9^, and deSUMOylation mediated by SENP1 in prostate cancer^10^.

A large-scale proteomic study of patients with breast cancer, leukemia and non-small cell lung cancer showed, that about 25% of the identified acetylation sites belonged to 12 most abundant serum proteins, including ALBU, A2M and complement factors, and the abundance of PTMs was different across studied cancer types^11^. Among modifications of plasma proteins, probably associated with the tumorigenesis, researchers note the role of alpha-1-antitrypsin (A1AT) nitrosylation in the regulation of apoptosis^31^, glycosylation and fucosylation of A1AT in ovarian and breast cancers^28^, fucosylation and sialylation of apolipoprotein C3 (APOC3) in colorectal cancer as well as fucosylation C3 complement in colorectal cancer^41^, and glycosylation of serotransferrin (TRFE) in oncology^42^.

Introduction of different PTMs can be caused by specific microenvironment of inflammation and necroptosis, and can alter protein functions^12^. Excessive fucosylation of C3 complement factor at N85 and sialylation at N939 patterns endorses the distinguishing of patient with colorectal cancer from those with adenoma, when changes in protein abundancy are not sufficient^13^. Specific glycoforms pattern has been proposed as utility in early diagnostic of hepatocellular carcinoma, despite the cancer-specific role of the detected glycosylation sited is not examined yet^14^.

Overproduction of reactive oxygen (ROS) and nitrogen species (RNS) may induce non-specific modification of plasma proteins, but the action on protein functions and structural stability is unequal. Nitrosylation of tryptophane in ALBU may significantly change its binding capacity^15^. At the same time, if TRFE is not affected significantly and does not reduce own iron-binding activity after nitrosylation, activities of CERU and A1AT are rather reduced^16^. Obviously, that undesirable local environment under pathological conditions may bring multiple untargeted PTMs, thus, causing unpredictable effects on protein functions and determine tumor progression.

In this report, we would like to highlight that breast and ovarian cancer-specific molecular events can be considered through the prism of post-translational actions and the PTM-induced conformational changes in protein structure. Structural biology has provided a good understanding of motif elements determined by the specific folding template of secondary structures. Such motifs are typically tightly packed and accommodated by the neighboring or closest segments of the polypeptide chain, helices, and β-strands, and maintain the originality of spatial folding regardless the homology of proteins, where such elements were being observed in^17,18^. Owing to the high stability of the protein globule, helical pairs are the most attractive for structural analysis. Therefore, in silico structural molecular analysis and modeling of PTMs introduction can be accomplished on isolated sustained helical pairs instead of ordered protein molecules, which improves calculations without losing of information.

We performed a structural analysis of the geometry and topology of motifs with PTM moieties accommodated in targeted peptides that derived from cancer-associated proteins. The study determined that 65 and 88 proteins in ovarian cancer and breast cancer groups, respectively, significantly differed in their abundance compared to the control group. We also assessed 74 and 25 proteins specific for ovarian cancer and breast cancer groups, respectively, and 50 proteins were shared between studied groups but misplaced in the control group. It has been found that the cancer-specific plasma proteome significantly is different from the aligned phenotype of healthy donors in particular localization of PTM moieties on the protein surface. Among the identified PTMs, 12 modified peptides were localized in eight proteins of ovarian cancer patients, whereas six modified peptides were localized in three proteins of breast cancer patients. It is supposed, that most oncopathologies can be induced by uncharacterized PTMs even before sensitive alterations in gene expression. Furthermore, based on the presented data, a particular PTMs pattern can be featured for a certain cancer phenotype.

## Results

### Proteomic analysis of plasma samples by ovarian and breast cancer

**Figure 1:**
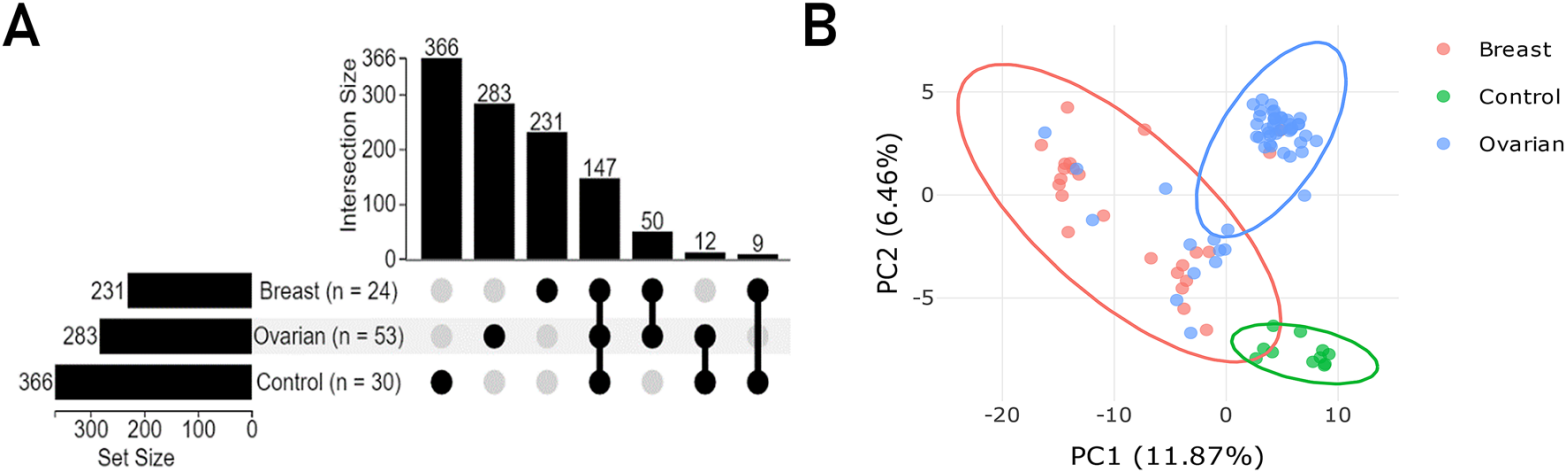
The upset diagram showing the distribution and the intercept size of protein identifications amongst groups of breast and ovarian cancer phenotypes, and the control group (**A**). PCA analysis carried out for the mutual fraction of proteins (*n* = 147) with no imputation and reflecting the quantitative alteration of the identified proteins between groups: breast cancer (BC)—red ellipse, ovarian cancer (OC)—blue ellipse and the control (CNT)—green ellipse. The abundancy (quantitative item) was fit to the log-scale (**B**).

Identification of plasma protein signatures that define ovarian cancer and breast cancer phenotypes was achieved by categorizing qualitative and quantitative features. The proteomic profiling revealed n = 231 and n = 283 proteins in breast and ovarian cancer groups, correspondingly, and n = 366 proteins found in the control group of healthy donors. Symmetry comparative analysis showed a wide cluster of proteins (n = 147) shared between all studied groups (including the control group), a smaller cluster for both types of cancer (n = 50; breast cancer and ovarian cancer), and two clusters distinct for patients with ovarian (n = 25) or breast (n = 74) cancer (Fig. 1A).

A comparative analysis of the protein content in blood samples of study participants revealed that 65 and 88 proteins (*p* < 0.05) in ovarian cancer and breast cancer groups, respectively, significantly differed in their abundance compared to those in the control group (Supplementary Table S1). Furthermore, the abundancy-based principal component analysis (PCA), taken for n = 147 proteins, returned satisfactory segregation of all three studied groups by the projection of the first two principal compounds with PC1 explaining 11.87% variance, and PC2 of 6.46% (Fig. 1B). Differentially expressed proteins with a *p*-value less than 0.05 and frequency more than 0.8 were assessed based on the measure of median abundancies ratio between groups of study and the control group. Totally, n = 65 differently expressed proteins with the mean frequency of 0.87 and n = 87 differently expressed proteins with the mean frequency of 0.95 were identified and estimated (Supplementary Table S1) in group with ovarian and breast cancer, correspondingly.

### Functional annotation

Among the mutual fractions of proteins detected and identified in all studied groups (n = 147), most were attributed as extracellular and involved in the activation of the complement system, including its terminal phase (n = 40, Pathways from Reactome External ID: R-HSA-166665, R-HSA-174577), platelet degranulation (n = 27, R-HSA-114608,), innate immune system (n = 49, R-HSA-168249), hemostasis (n = 37, R-HSA-109582), and regulation of post-translational phosphorylation (n = 18, R-HSA-8957275). Proteins shared between the cancerous groups are also characterized as extracellular and regulate the immune response (n = 10, R-HSA-168256), metabolism of proteins (n = 10, R-HSA-392499), innate immune system (n = 8, R-HSA-168249), and hemostasis (n = 10, R-HSA-109582). Cancer-specific proteins are implicated in vesicle-mediated transport (n = 11, R-HSA-5653656), activation of the immune system (n = 35, R-HSA-168249, R-HSA-168256), and membrane trafficking (n = 8, R-HSA-199991).

### Post-translational modifications of proteins

Modification moieties were analyzed for the five most prevalent types of PTM, including phosphorylation of serine (pS), threonine (pT), tyrosine (pY), N-terminal acetylation of lysine (ac-K), and ubiquitination of lysine (Supplementary Fig. S1). It should be noticed, that while ubiquitination is the most prevalent PTM in the control group (up 61% among the compete study population), it represents the relatively small input in patients with ovarian cancer (32%) and the least contribution (only 7%) in the group with breast cancer (Supplementary Fig. S1), which possibly might indicate significantly suppressed process of proteins degradation in groups with cancer. Instead, acetylation has been tracked as the most abundant PTM in groups with cancer (36% and 56% in breast and ovarian cancer, correspondingly), which might be caused by the ongoing proliferation, chromatin remodeling and immune signal transduction, which are actively dependent on acetylation uptake in the regular enzyme-dependent manner. In oppose the contribution of acetylation among healthy donors was marginal and made only 8% (Supplementary Fig. S1). PTMs were extracted into a separate list after percolation and manual curation of spectra of PTM-carrying peptides that match at least 80% sequence coverage, and carrying properly defined b/y-ion pairs (Table 1). We observed PTMs among 12 peptides derived from eight proteins in patients with ovarian cancer and six peptides, carrying modified moieties, from three proteins found in breast cancer patients.

**Table 1:** The list of proteins carrying the defined PTMs and found specifically in patients with ovarian cancer or patients with breast cancer.

Gene	Protein	Sequence of peptide carrying PTM	PSM	Number of peptide identifications	Seq., %	B/Y
**PTMs observed in patients with ovarian cancer (OC)**						
A1AT	Alpha-1-antitrypsin	**ac(K)***-*QINDYVEK	131	17	55	1B/8Y
		TDTSHHDQDHPTFN**ac(K)**ITPNLAEFAFSLYR	131	17	55	0B/6Y
ALBU	Albumin	EQL**ac(K)**AVMDDFAAFVEK	94	17	32	0B/7Y
		NYAEA**ac(K)**DVFLGMFLYEYAR	454	25	31	0B/6Y
		RHPYF**p(Y)**APELLFFAK	618	46	76	3B/6Y
		VFDE**ac(K)**PLVEEPQNLIK	618	46	76	0B/5Y
APOA2	Apolipoprotein A-II	EPCVE**p(S)**LVSQYFQTVTDYGK	29	6	63	1B/6Y
CO3	Complement C3	**ac(K)**VLLDGVQNPR	161	32	31	0B/5Y
HV307	Ig heavy chain V-III region CAM	DDS**ac(K)**NTLYLQMNSLR	11	4	12	0B/9Y
IGHA 1	Ig alpha-1 chain C region	SGNTFRPEVHLLPPP**p(S)**EELALNELVTLTCLAR	90	13	63	0B/7Y
KNG1	Kininogen-1	E**p(T)**TCSKESNEELTESCETK	13	9	21	7B/9Y
TRFE	Serotransferrin	MDA**ac(K)**MYLGYEYVTAIR	145	19	30	0B/8Y
**PTMs observed in patients with breast cancer (BC)**						
ALBU	Albumin	EQL**ac(K)**AVMDDFAAFVEK	465	27	43	1B/7Y
		**ac(K)**VPQVSTPTLVEVSR	465	27	43	0B/5Y
		NYAEA**ac(K)**DVFLGMFLYEYAR	687	48	79	0B/8Y
APOA1	Apolipoprotein A-I	QLNL**ac(K)**LLDNWDSVTSTFSK	43	16	73	4B/10Y
TRFE	Serotransferrin	**ac(K)**SASDLTWDNLK	357	38	66	0B/9Y
		MDA**ac(K)**MYLGYEYVTAIR	159	17	34	2B/8Y

Bold values indicate the exact affected (modified) amino acid residue to attract readers' attention and for easier navigation.

Peptides are listed with the main accompanying mass-spectrometric characterizations: PSM—peptide spectra match (is the number of spectra matching the theoretical peptide sequence with high score); b- and y-type of fragment ions are C- and N-terminal sequential fragment ions populated after peptide (precursor ion) decay. Complete results are available in the Supplemental Materials Table S2.

*Ac(K)* acetylation of lysine; *p(Y), p(T), p(S)* phosphorylation of tyrosine, threonine and serine, correspondingly; *PSM* peptide spectra; *Seq., %* amino acids coverage for the identified peptides; *b/y* the number of revealed and attained b- and y-type fragment ions.

There were also a few overlapping PTMs revealed in albumin and Ig heavy chain V-III region CAM. Sequence coverage of peptides carrying PTMs consisted of 12% to 79%, and at least three distinct and proteotypic peptides were identified with a high confidence score for each protein. A high scoring function for the identified peptides was provided by satisfying the number of b- and y-type ions (Supplementary Materials Table S1) with marginal measurement error.

**Figure 2:**
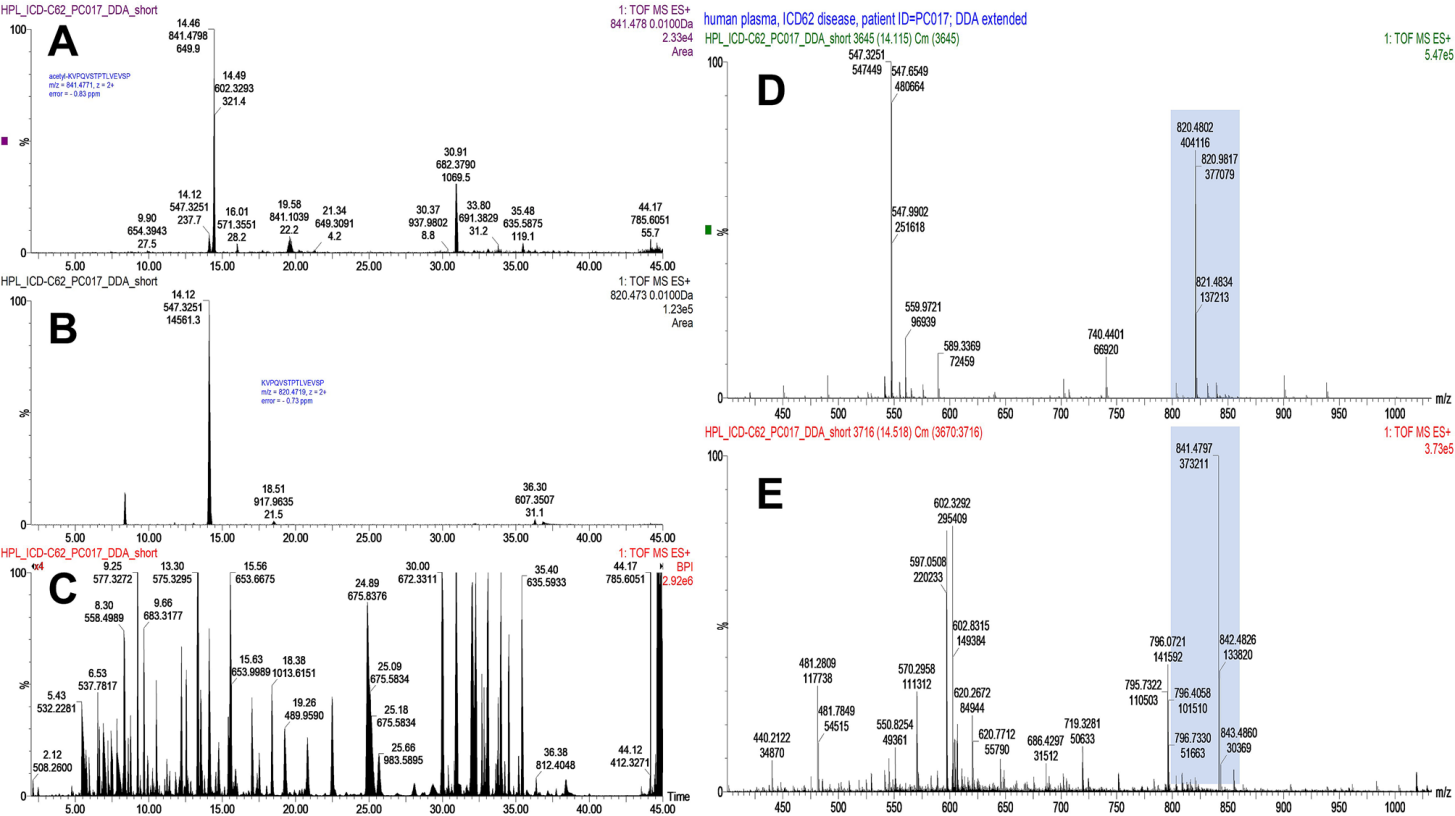
Overview of the targeted mass spectrometry analysis (t-MS2) exemplified on peptide KVPQVSTPTLVEVSR (ALBU) in patient with breast cancer. Extracted ion chromatograms of the modified peptide with *m/z* = 841.4774^2+^, mass error is -0.83 ppm detected at 14.46 min (**A**) and its intact (unmodified) counterpart peptide with *m/z* = 820.4719^2+^, mass error is -0.73 ppm, detected at 14.12 min and shadowed by the unknown peak with *m/z* = 547.3251 (charge state z = 2 +) (**B**). Base-peak chromatogram resulted after targeted MS2 analysis shown within analytical time range from 0 to 45 min and 4-folds magnified in intensity scaled (**C**). Averaged at half of maximum intensity mass spectra of the intact peptide (**D**) and modified peptide (**E**). Base peak with *m/z* = 547.3251^2+^ is visible on the averaged spectra of the intact peptide (**D**).

Targeted proteomic analysis was conducted to validate the presence of modified peptides and their intact (unmodified) counterparts, which have been detected over the mass spectrometric discovery analysis (Fig. 2). Although, the detected peptides carried PTMs at previously non-reported locations, the existence of intact peptide lacking the modifying group is highly expected. The result of targeted MS analysis demonstrated that all peptides originated from patients with ovarian or breast cancer can be found in both modified and intact conditions (Fig. 2 and Supplementary Fig. S2) and fragmentation spectra satisfies coverage of PTM of interest (Supplementary Fig. S2).

### Structural analysis of proteins carrying PTMs

Several well-known structural motifs are characterized by specific spatial folding and geometry in structural biology. Such motifs are typically tightly molded and shaped by the adjustment segments of polypeptide chains, helices, and β-strands^17,18^.

In this study, we investigated motifs molded by secondary structures and carrying different PTMs. Such motifs are represented as helical pairs designed by two consequent helices bridged with irregular connections of different lengths and conformations^17^. According to the geometry rules and topological classification of helical pairs (Supplementary Fig. S3), such motifs can be classified on α-α-corners, α-α-hairpins, and L- and V-structures, and modifying moieties are frequently observed within these structures. We selected protein structures from the Protein Data Bank (PDB) matching the targeted peptides that carry empirically identified PTMs^17^. Each selection sampled from 1 to 172 protein structures fitting the recognition of specific polypeptide chain rules (Table 2, column “PDB structures”).

**Table 2:** Solvent accessible area alterations of amino acid residue and its active environment before and after modification (mounting of PTM) in breast and ovarian cancer-specific proteins.

Gene (Protein name)	Sequence of peptide carrying PTM	Secondary structure localization	PDB structures	Average area accessible to the solvent, Å^2^				Helices	Motives
				Amino acid		Active environment			
				Intact	PTM	Intact	PTM		
**Ovarian cancer**									
A1AT (Alpha-1-antitrypsin)	**ac(K)-**QINDYVEK	Helix	35	119,99	167,54	335,14	364,17	4	1
	TDTSHHDQDHPTFN-**ac(K)-**ITPNLAEFAFSLYR	Connection	23	109,78	153,36	346,66	377,32		
ALBU (Albumin)	EQL-**ac(K)-**AVMDDFAAFVEK	Helix	156	113,03	135,71	441,31	431,30	30	4
	**ac(k)-**VPQVSTPTLVEVSR	helix	172	21,5	37,29	320,08	312,11		3
	NYAEa-**ac(K)-**DVFLGMFLYEYAR	Connection	162	82,02	108,97	294,16	305,17		3
	RHPYF-**p(Y)-**APELLFFAK	Helix	163	22,49	31,01	165,83	154,03		3
	VFDEF-**ac(K)-**PLVEEPQNLIK	Helix	162	131,78	179,83	278,90	316,22		2
APOA2 (Apolipoprotein A-II)	EPCVE-**p(S)-**LVSQYFQTVTDYGK	Helix	56	69,47	101,31	498,39	501,38	4	n/d*
CO3 (Complement C3)	YF-**ac(k)-**PGMPFDLMVFVTNPDGSPAYR	Helix	51	13,86	16,87	134,78	110,5	1	n/d
HV307 (Ig heavy chain V-III)	DDS-**ac(K)-**NTLYLQMNSLR	Connection	2	126,05	177,7	316	349,85	0	0
IGHA1 (Ig alpha-1 chain)	SGNTFRPEVHLLPPP-**p(S)-**EELALNELVTLTCLAR	Helix	34	103,55	168,88	318,55	361,30	3	1
**Breast cancer**									
ALBU (Albumin)	NYAEA-**ac(K)-**DVFLGMFLYEYA	Helix	162	82,02	108,97	294,16	305,17	30	3
	EQL-**ac(K)-**AVMDDFAAFVEK	Helix	156	113,03	135,71	441,31	431,30		4
APOA1 (Apolipoprotein A-I)	QLNL **ac(K)-**LLDNWDSVTSTFSK	Helix	6	136,75	184,78	317,5	348,78	8	n/d
TRFE (Serotransferrin)	**ac(K)-**SASDLTWDNLK	Helix	32	111,32	155,53	322,75	349,13	22	12
	MDA-**ac(K)-**MYLGYEYVTAIR	Connection	72	64,48	104,60	260,24	279,48	22	12

Bold values indicate the exact affected (modified) amino acid residue to attract readers' attention and for easier navigation.

n/d* – motives with interacting helices are not found; PDB structures – the number of PDB structures matching the target polypeptide chain; PTM SEQQ – amino acid sequence of polypeptide chain carrying the detected and identified PTM.

The PTMs that we have identified in our study are localized in stable super-secondary motifs, and the majority of them are exhibited in helices, while only a small portion of PTM-carrying peptides is localized in connections (Table 2, column “Secondary structure localization”). Notable, that majority of peptides containing the acetyl-group are allocated within helices, excluding peptide of TREF protein in ovarian cancer and several long-length peptides of A1AT, ALBU and variable domain of Ig-heavy chain in breast cancer group (Table 2). Generally, it falls with the previously defined localization of acetyl moieties in the regular secondary structural elements^19^. Besides, supersecondary motifs, in which PTMs have been identified, are always exposed to the solvent.

A comparative analysis of the geometric features of the assayed protein structures was performed according to several criteria, including similarity of defined helical pairs among selected polypeptide chains and the solvent accessibility to the local folded motif. In addition, the type of spatial conformation for motifs shaped from selected secondary structures and the influence of modified amino acid residues on protein structure stability were also accounted.

Molecular dynamics experiments determined the stability of ovarian and breast cancer-specific protein structures bearing PTM moieties. The solvent-accessible surface area of the intact amino acid residue exposed to the solvent was always less than that of the modified residue (Table 2, column “Active environment”). Thus, the modified amino acid residues are continuously exposed to the solvent, and the modification process is associated with the enlargement of the solvent-accessible surface area. The surface area of the modified residue exceeded thoset of the intact (unmodified) amino acid residue by 50%.

The adjacent environment (i.e., neighboring amino acid residues) was evaluated as an alteration of the total surface area accessible to the surrounding solvent (Table 2, column “Active environment”). The mean surface area of the modified environment frequently exceeded that of the intact (unmodified) environment. However, the difference is not as explicit as for separate modified amino acid residue. In some cases, the PTM moiety can significantly increase the accessible surface area. However, the total surface area of the exposed active environment is equal to or even less than that of the unmodified polypeptide chain. Hence, it can be assumed that the neighboring amino acid residues eliminate surface area increased after the PTM introduction.

**Figure 3:**
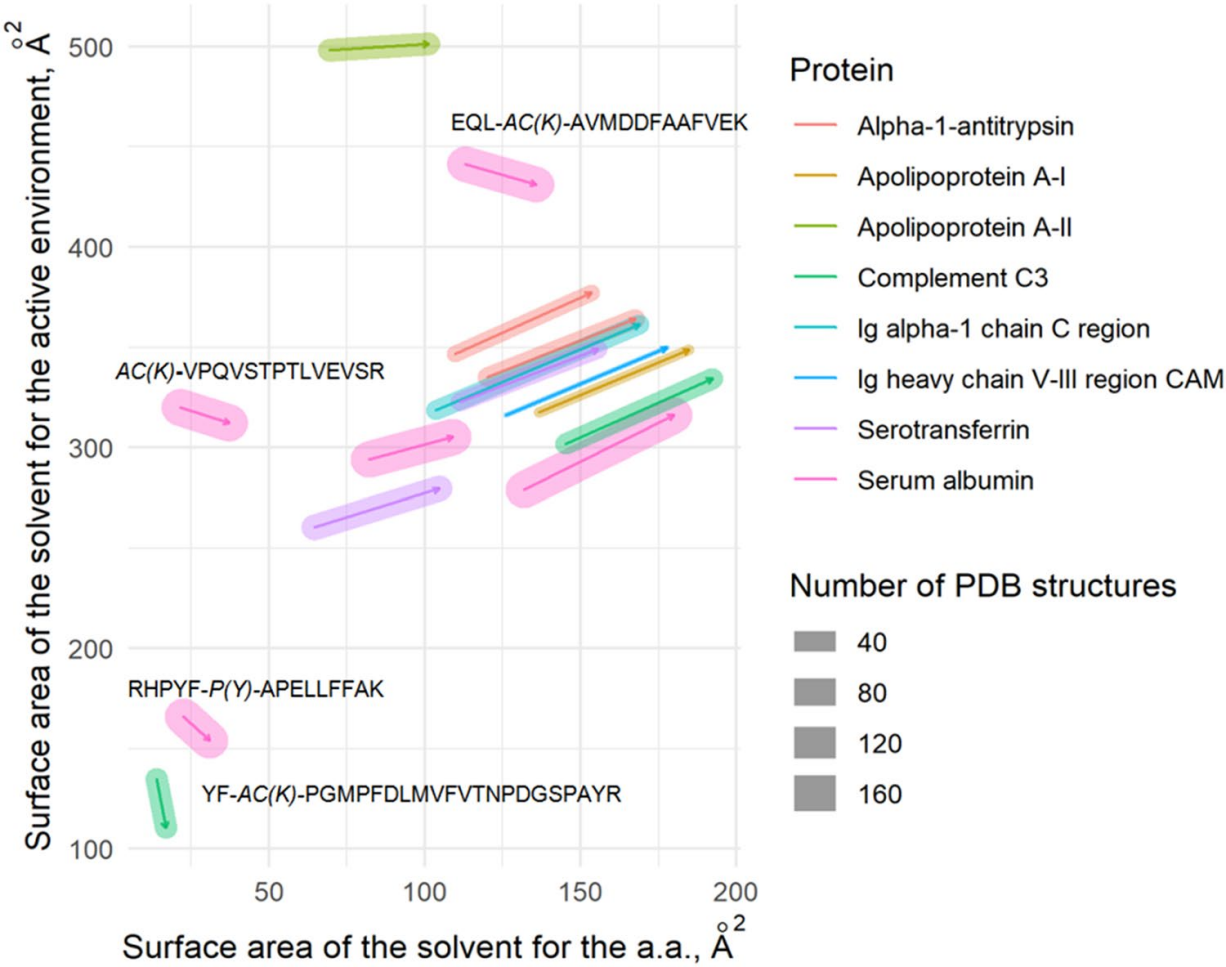
Variation of the total solvent-accessible area (in square angstroms; Å^2^) of active environment after modification of amino acid residue (mounting of PTM). The modified proteins found in plasma samples of patients with breast cancer and ovarian cancer are color-coded. The number of PDB matched structures used for the analysis are designated by bar size.

The active environment is defined as the amino acids surrounding the modified residue and is capable of changing the solvent-accessible surface area of certain motif. We determined the active environment within each motif and identified the number of constituent amino acids and their spatial coordinates within the affected polypeptide chain (Fig. 2). Excluding four cases, the total solvent-accessible surface area of the modified residue and its active environment increased, in contrast to that for the intact residue. The notable instances (peptides EQL-***ac(K)***-AVMDDFAAFVEK (ALBU), ***ac(K)***-VPQVSTPTLVEVSR (ALBU), RHPYF-***p(Y)***-APELLFFAK (ALBU), and YF-***ac(K)***-PGMPFDLMVFVTNPDGSPAYR (CO3)) are highlighted by the larger solvent-accessible surface area of the modified residue compared to the intact residue (Fig. 3). However, the solvent-accessible surface area of their active environment is smaller compared to unchanged residues.

**Figure 4:**
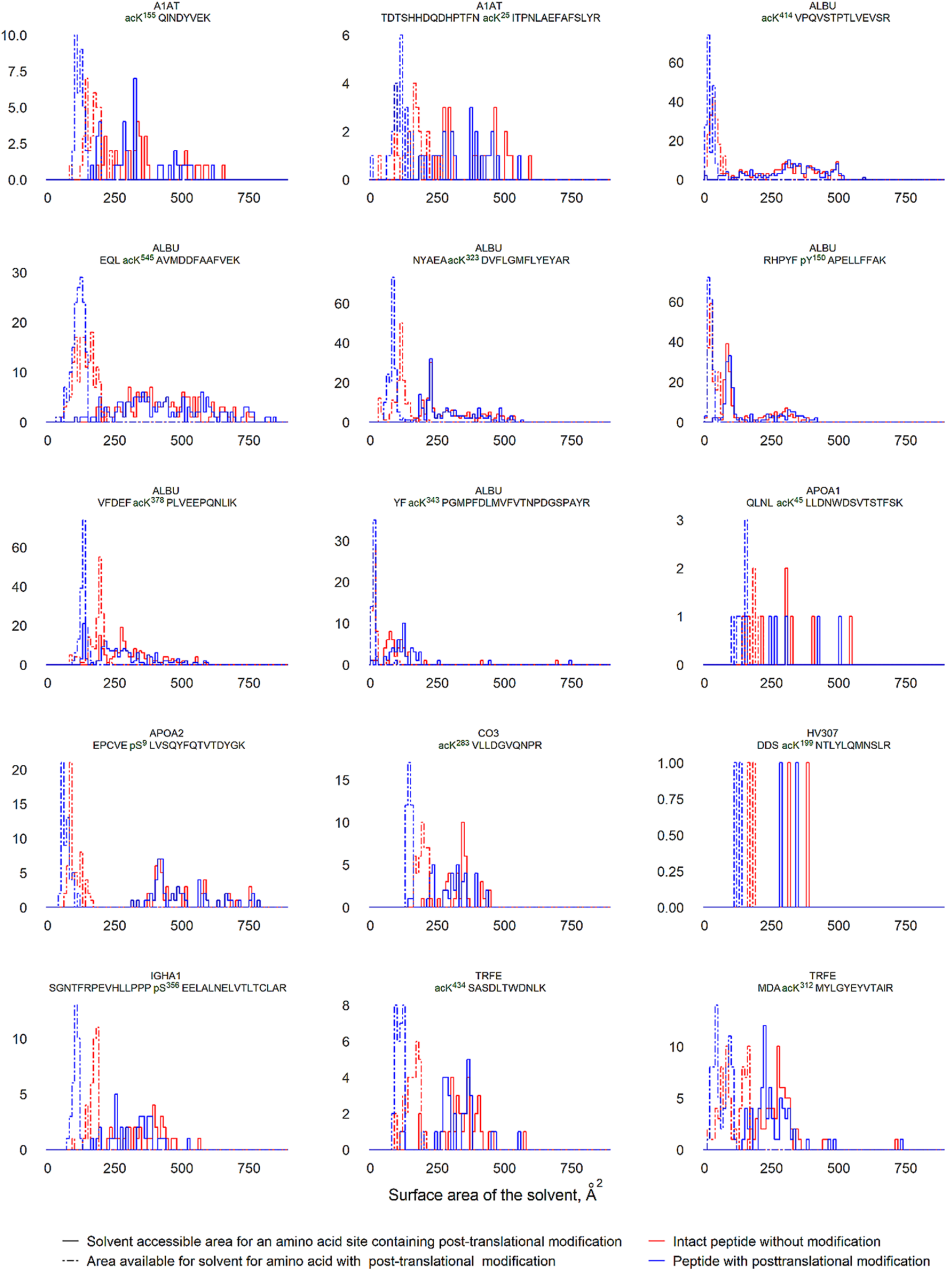
The distribution of proteins population with the identified PTMs depending on the solvent-accessible area for the certain amino acid residue and its active environment before and after mounting of PTM. The horizontal axis indicated the surface area, accessible for the surrounding solvent (in Å^2^); the vertical axis indicated the number of the affected protein molecules. Color-code defines amino acids before modification (blue dashed line), amino acids after modification (pink dashed line), active environment before modification (blue solid line) and active environment after modification (pink solid line).

The distribution of protein population bearing the identified PTMs depends on the solvent-accessible area for the particular amino acid residue and its active environment before and after the introduction of PTM (Table 2). The modifying moiety increases exhibition of the surface area of the corresponding amino acid residue to solvent and its active environment (Fig. 4). However, the summed solvent-accessible surface areas do not always exceed those before the modification and are characterized by dense scattering.

### Molecular dynamics simulation of protein molecules containing PTM associated with the development of ovarian and breast cancer

In this study, we analyzed the similarity, stability, and influence of PTM moiety on the local solvent-accessibility area alteration in the modified polypeptide chain of protein molecule. Results of molecular dynamics showed that the geometry and topology features of motifs before and after the PTM mounting are kept within acceptable ranges over the time of the simulation experiment. Particular attention has been drawn to motifs with strongly interacting and axially intercepting helices, typically, α-α-corners. The between-distance is negligible (contacting helices), and the area and projection perimeter are distinct from the null value. We did not scrutinize L- and V-structures because helices do not intercept (d ≠ r, d < r) in the instance, and the area and perimeter for such motifs are close to a null value. Simultaneously, the contribution of motifs comprising of more than two helices allowed to extend the range of observable structures. The main criterion for inclusion in the MD (molecular dynamics) simulation was the actual contact between helices; however, the connection length and its structure did not act as a significant input.

**Figure 5:**
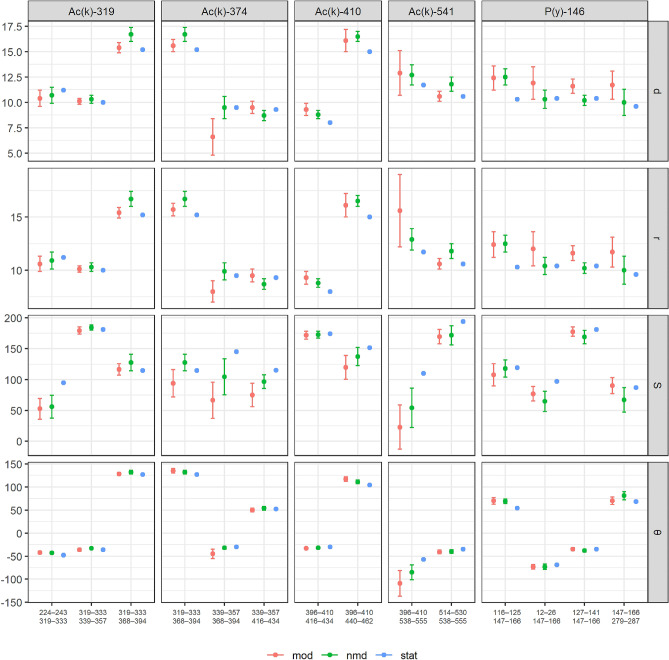
Calculated geometrical features of albumin (ALBU) motives with PTM moiety. Each motif is characterized by the estimated geometrical features for intact molecule (*stat*; blue color), calculated results of molecular dynamics for the unmodified molecule (*nmd*; green color), and calculated features for the modified molecule (*mod*; red color). The right axis indicates (d) distances between helices, (r) inter-planar distance between helices, S—area of the spatial projection, and θ—torsion angle.

By employing these selection criteria, we expanded the number of motifs that can be sampled for the analysis. In the previous study, only helices pairs comprising two sequential helices were utilized. In this study, we investigated closely contacted helices (from 1 to 30 helices) with intersecting axes. Simultaneously, the connection may vary in length and conformation and may include secondary structures (Table 2, column “Motifs”). We detected five PTM moieties in albumin (ALBU), three in IGHA1 and CO3, four in APOA2 and A1AT, and eight in APOA1 (Supplementary Table S2 and Fig. 5). The calculated coordinates for the detected PTMs, α- and θ-angles, minimal (r), and inter-planar (d) distances between helices, areas, perimeters, and their standard deviations (sd, sr, sα, sθ, sS, and sP) are presented in Supplementary Table S2.

The result of molecular dynamics experiments showed that during the set time (0.5 ns), the geometrical features of studied motifs (intact and modified) were within the acceptable ranges. However, we have identified conformational changes that distinguish modified from intact forms. It was demonstrated that the stability of structural motifs directly depends on the strength of interactions between helices (as indicated by values of d, r, S, P, etc.). It has been observed that the introduction of PTM moiety does not induce complete rupture between interacting helices and, consequently, does not disrupt the motif with the accommodated PTM moiety (Fig. 5).

Although mounting of the modifying moiety did not lead to the complete rupture of intramolecular bonds and motif disruption, but some protein molecules showed an ambiguous attitude (Fig. 5 and Supplementary Table S2). Two distinct motifs containing polypeptide chain EQL-***ac(K)***-AVMDDFAAFVEK with acetylated lysine should be noted. The topology of the first modified helix was 396–410 (400–414 in the intact molecule), and the location of the second modified helix was 538–555 (542–559 in the intact molecule). The motif comprises seven helices, although there are five helices between the considered contacting helices and the intersecting axes. At the beginning of the molecular simulation, the initial geometry met the following features: the minimal and inter-planar distances were equal and made 11.7 Å, while the area and polygonal perimeter were 110.2 Å^2^ and 43 Å, respectively, and the axial angles were α = 55° and θ = − 57°. Following-up the molecular simulation, the distances (r and d) increased, indicating misalignment between helices. The axial angles, area, and perimeter also decreased. The inspected predisposition held for both modified and unmodified motifs; however, the modified structure inclined to a more remarkable alteration in conformational geometry (inter-planar and minimal distances were 12.9 Å and 15.6 Å; area and perimeter were S = 22.9Å^2^ and P = 12.1 Å, correspondingly; torsion angle θ = − 109°).

A counter situation was observed for the motif with two consequent helices localized at positions 514–530 and 538–555 (positions 518–534 and 542–559 in the unmodified molecule). Initially, the motif was attributed to the following customized settings: d = r = 10.6 Å, S = 194.2 Å^2^, P = 59.6 Å, α = 41°, θ = − 35°. The geometry changed insignificantly during the molecular simulation and made d = r = 11.8 Å, S = 171.8 Å^2^, P = 55.5 Å, α = 46°, θ = − 40° for the intact molecule; and d = r = 10.6 Å, S = 169.8 Å^2^, P = 55.4 Å, α = 50°, θ = − 41° for the molecule with the introduced PTM moiety, which indicates higher stability than the previously examined helical pair.

## Discussion

Plenty of reports demonstrated, that the majority of constituent post-translational modifications are essential for the regulation of protein functional activities. The exact effect may vary, depending on the context, and can follow to enhancement or, oppositely, complete abrogation of catalytic, binding or transducing activities. Some PTMs, especially acetylation and phosphorylation, are responsible for delicate guiding of genes expression, which makes them high-potency targets if consider anti-cancer therapy and investigation of tumor invasion and progression. Non-specific, or previously not being reported, PTMs are frequently acquired during the proteomic assay of cancer cells, but their impact on protein properties and structural stability typically remains under assumption^20,21^. It is especially difficult to investigate protein structure flexibility, if multifunctional protein can put on many different modifications (phosphorylation, acetylation, ubiquitination, SUMOylating and GlcNAcylation) depending on the contextual conditions^22^ Hence, the distinguishing of regular or pathological “PTM code”, or PTM signature, is a topic of growing interest. We hypothesize that the discovery of PTMs signature may become a central point for a deeper understanding of signals transduction and metabolic transformations in cancer cells.

In this study, we attempted to specify PTM-induced conformational changes in proteins found in patients with ovarian or breast cancers. We suggest that such PTMs, whether mounted at an unspecific topology or cause conformation instability, can change and modulate protein activities and functional properties. Accumulated evidence exists regarding the cautioning role for such PTMs as master epigenetic players, providing an excellent opportunity for the utility of PTM patterns^22^, including specifically recognized for certain cancer phenotype^23^.

Recently, it has been demonstrated that different types of PTM can be specifically distributed depending on protein functional category or its subcellular localization^24^, which indicates accumulation of type-specific PTMs in certain cancer phenotype and support a clue in tumorigenesis. Similarly, we observed a sensible variation of PTM types across cancer phenotypes in our study (Supplementary Fig. S1)^11^.

Due to cancer is typically accompanied by the immune response and acute inflammation, alpha-1-antitrypsin (A1AT) is one of the most scrutinized markers of tumorigenesis. Because of its vigorous conformational flexibility, A1AT can take on different forms (polymeric, oxidized, cleft) adapted for specific biological processes and ligand types^25,26^. Reports indicate that PTMs introduction induces A1AT conformational changes, modulating structural sustainability and managing catalytic activities^27^. Such PTMs enhance protein stability and sterically protect the molecule against proteolytic activity, degradation and conformation-induced aggregation, thus increasing the protein half-life^28–30^. Recent studies definitely characterized different glycosylation patterns for patients with non-small-cell lung cancer and lung adenocarcinoma, and such patterns may serve as a promising marker for non-invasive differentiation and early diagnostics^31^. Meanwhile, unspecific bi-antennary di-sialylated glycosylation of A1AT has been demonstrated in patients with aggressive form of ovarian and breast cancers and assumingly may contribute in rapid malignancy due to partial inactivation of A1AT^32^.

In this study, we discovered two novel PTMs (ac-K^25^ and ac-K^125^), which are distant from the known modifiable sites and sites of interaction with different endogenous ligands (Tables 3 and 4). The propensity for PTM-induced conformational polymorphism emphasizes a comprehensive pattern of A1AT molecular functions. It is suggested that proteins acetylation is tightly associated with the regulatory function and more frequent for proteins with dual extra- and intracellular localization^33^, whereas proteins involved in inflammation readily accumulate different untargeted PTMs. However, most of such PTMs has no significant damaging effect on the protein structure. Thus, although two newly discovered PTMs of A1AT do not change the protein structure dramatically, assumingly they can bear unknown functions and possess a high potency in the modulation of immune reactivity.

**Table 3:** Functional amendments of proteins under the impact of the selected PTMs reported in the literature and observed in the present study.

Protein	Our experiment	Biological role of protein	PTM, literature data	Biological role of the allocated PTM	Refs
A1AT	ac-К^25^, ac-К^125^	Tissue protection from the hydrolytic catalytic activity	Glu-К^342^ и Glu-V^75^	The dire role of polymers aggregation is uncertain but observable	^65,66^
			Ox-M^351^ и ox-M^358^	Signs of oxidative stress and pro-inflammatory activity	^67^
			NO-C^232^	Regulation of apoptosis	^68^
			Galactosylation and Fucosylation patterns	Specific distinguishing of non-small-cell lung cancer from lung adenocarcinoma	^31^
			3 N-glycosylation sites	Increase outer arm fucosylation in ovarian cancer and breast cancer patients	^69^
			Carbamoyl-K^359^	Enhances autoimmune response in patients with rheumatoid arthritis	^32^
ALBU	ac-К^545^, ac-К^323^, ac-К^343^, ac-К^378^	Regulation of affinity binding with various low-molecular-weight ligands	Glu-C^34^	Reduction of affinity binding with tryptophane, bilirubin, warfarin, and diazepam	^70^
			NO-C^34^	Regulation of organic anions and metal transportation; reduction of affinity binding with fatty acids	^37^
			SH-C^34^	Marker of oxidative stress	^71^
APOA2	p-S^9^	Lipid binding and homeostasis^11^	N/D	N/D	^72^
CO3	Ac-K^283^	May act as pro-oncogenic factor due to positive regulation of VEGF expression level; involved in angiogenesis, organization and clearance of extracellular matrix, and cell migration^12^	Glc-Asn^917^ (α-chain); Glc-Asn^63^ (β-chain)	N/D	^73^
			p-T^1009^ (intact C3)	N/D	^74^
			Fuc-N^85^ and Sia-N^939^	Increase in α-2,6-sialic acid and in fucosylation in colorectal cancers patients	^13^
TRFE	Ac-K^331^, Ac-K^453^	Binding and transfer of iron ions; regulation of iron level, especially during pregnancy	High level of glycosylation of N^432^ and N^630^	Acute inflammation in pancreatic patients in advance to tumorigenesis	^75^
KNG1	p-T^326^	Regulation of blood coagulation, blood glucose level and induction of nociceptors	Non-specific glycosylation of N^294^	Unknown role; detected in colorectal cancer patients	^13^

**Table 4:** List of known interaction sites deposited in PBD protein structures, located in close proximity to post-translationally modified amino acid residues found in patients with breast and ovaria cancer.

Protein entry name	PDB ID	Ligand	Locus of interaction	References	Structure illustration
A1AT	6HX4	Fab-fragment	R199–Q215 and S362–K368	^76^	
	7NPL	Glycerol	V374 and L251	^77^	
	7NPK	Glycerol	S118 and L51	^77^	
	1EZX	Trypsin	S290–M335	^78^	
	1OPH	Trypsinogen	G349–P361	^79^	
	3CWM	Citrate	L241 and L288	^80^	
	7AEL	Sulfate anion	L298	^81^	
ALBU	3B9M	Myristate	A158 and R186	^82^	
	4Z69	Palmitic acid	L345, C437 and A449	^83^	
	2ESG	IgA1	P35–R144	^84^	
	2VUE	4Z,15E-bilirubin-IX-alpha	R114, P127 and L182–K190	^85^	
APOA1	3R2P	Homodimer	R84–E111 and H162–A181	^86^	
	3K2S	Phospholipids	Whole seguence	^87^	
TRFE	3S9N	Transferrin receptor protein	L86–N90, R338–G343 and S362–N375	^88^	
	3S9M	Iron-cation	L242	^88^	

Blue color indicates sites of interaction with ligands, red color designates amino acids with PTM found in our study.

Obviously, that PTM falling the functional region or in close proximity to catalytic center may have the most profound effect on activity. In this context, albumin draws an attention due to its ability to transfer and bind with a wide variety of functional molecules, including exogenous origin^34^. There is evidence of many albumin modifications with vague responsibilities, but changing its affinity. Glycation of Cys^34^ is the most examined and known albumin PTM, that induces conformational change and favors the binding capacity with various pharmaceutical ligands, including warfarin, tolazamide, acetohexamide, and tolbutamide^35,36^. In contrast, S-nitrosylation of Cys^34^ is responsible for transporting of anionic organic compounds and heavy cations and decreases protein affinity to fatty acids^37,38^.

Previously, we have recognized several PTMs in albumin (acetylation and phosphorylation) in patients with colorectal cancer^17^. We hypothesized that phosphorylation at the warfarin-binding site (Cys^34^) might be caused by dysregulation of cell signaling network and contribute meaningfully to tumor growth and progression^17^. The presently discovered PTMs (Table 4) may act as regulators of the affinity to low-molecular-weight compounds, since they are exposed at structural domain II (topological position 196–383) and domain III (topological position 384–585) closely localized to the fatty acids (S342 and R348) and drugs (R348-E450) binding sites^39^. Therefore, the effect of such PTMs can be extended to the system-level scale, where the modified ALBU can improperly interact with its partners, thence, affecting different signaling pathways in a bypass manner.

Numerous studies reported that phosphorylation of some resident serum proteins is up-regulated in ovarian and breast cancer patients^20,40^ and the complement system plays a central role in cancer cells control and sensitivity. Being a part of the innate immune response, the complement system can be initiated in three different pathways, of which the classical pathway is triggered after binding with either circulating or surface-immobilized immune-complexes^41^. Due to supreme influence on angiogenesis, positive regulation of VEGF, cell migration, and extracellular matrix reorganization, the C3 factor is frequently mentioned beyond the immune system but in the context of malignancy origination and oncogenic potency^42^.

Recently, the phosphorylation of pT^1009^ has been detected as a new yet uncharacterized PTM, apparently produced following the catalytic processing of the cleaved C3-factor by kinases^43^. In our study, the attendance of pT^1009^ was also established in patients with breast and ovarian cancer, and completely lacked in the control group (Table 3). The C3-factor can be phosphorylated at numerous different sites, which expectedly affect its structural properties and control functional activity; however, the consequence of such PTMs introduction is uncertain due to insufficient attention to the role of C3 phosphorylation in cancer pathophysiology. We suggest the high oncogenic potency of the recognized PTM moiety because the phosphorylation of pT^1009^ was frequently detected in this and other studies of cancer patients^40^. Besides, we observed another cancer-specific modification (Ac-K^283^), which induces a drastic conformational change in C3 molecule, according to the molecular dynamic simulation, but keeps the C3-factor stable, which probably reflects the acquisition of new activities and functional properties. We assume, that the newly gained PTM-induced properties might be related to the tumor-promoting effect of complement cascade activation and promote the known phenomenon of the complement-caused increase of metastasis^44,45^.

Changes of complement system status is frequently observed in patients with cancer, but changes in iron status was found to be the most prevalent in patients with advanced cancer. It is suggested, that the cancer-related anemia can be caused by chronic inflammation or as an effect of treatment^46^. Previously, it has been shown that serotransferrin (TRFE) is the most affected plasma protein in patients with adenocarcinoma and especially altered after chemotherapy treatment^45^. The PTM machinery produces several proteoforms of serotransferrin thereby contributing in the disturbance of iron status^47^. In this respect, changes in the expression of serotransferrin and its receptors might be targeted for the anti-cancer therapy. It was debated, that cancer patients are characterized by the distinct glycosylation patterns of TRFE, which may influence the binding capacity and restrict interaction with receptors of TRFE^48^. Although, numerous sensitive glycosylation sites in TRFE are rigorously investigated in cancer phenotypes, their versatile roles remain mostly suggestive and outcome from the iron transporting function of the protein. We identified two new PTM sites in TRFE at Ac-K^331^ and Ac-K^453^ in close proximity to the receptor interacting site (Table 4). It has been examined that despite introduction of these PTMs, the stability of TRFE is still adequate, however the Ac-K^453^ is the closest site to iron-binding Y^445^ residue and the presence of acetyl-moiety may prevent tight interaction of the second iron ion, hence, limiting binding capacity of TRFE.

In so far proteins responsible for binding and carrying of various ligands are the primary target in cancer therapy, combination of apolipoproteins is supposed appropriate signature in disease diagnostics and treatment response monitoring. Apolipoproteins (APO) bind lipids and act as ligands for cell surface receptors and co-factors for several enzymes^49^. Alterations of different apolipoprotein family members are also revealed in many unrelated to cancer pathologies^50–52^, which makes such proteins hard to use. Patients with breast cancer are distinguished by methylation and hydroxylation patterns of APOE, which may contribute greatly in breast cancer progression^21^. Meanwhile, it has been reported, that APOA2 bears different types of PTM (phosphorylation and acetylation) within initiation center of APOA2 homodimerization in patients with breast and ovarian cancer (Table 4), which meaningfully prevents proper functioning and embedding of APOA2 into HDL particles. However, the possible oncogenic role of modified or intact form of APOA2 is still unclear, since its expression level is frequently controversial in different cancer types (Table 3).

In summary, the irregular pattern of different PTM types is frequently observed in cancer phenotype, so it seems on the surface that PTMs are tightly associated with tumorigenesis and opportune metastasis. Quite a lot of targeted studies showed, that such non-specific PTMs exhibit regulatory properties and may influence significantly on the protein structure. However, the total effect on proteins structure stability is not so dramatic and does not disrupt the protein globule, so assumingly, newly introduced PTMs modulate protein activity. The question of whether such “non-specific” PTMs are the defense mechanism against cancer or, oppositely, the offense strategy of cancer cells is rather open due to many controversial reports and, probably, will be unsolved for a while until the study of these PTMs will grow out of infancy.

## Conclusion

In this study, we reported PTMs found at non-specific localizations on molecular surfaces that have never been annotated previously and not detected in normal plasma or regular cellular proteins. We used a combination of discovery proteomics and molecular dynamic simulation approaches for analyzing conformational fluctuations and structural re-ordering caused by the introduction of post-translational modifications. Proteomic analysis revealed uneven prevalence of different PTMs across breast and ovarian cancer phenotypes, and the control group.

Structural analysis targeted a small fragment of the protein molecule (motif) and the active environment organized by adjacent amino acid residues. The examined PTMs are located within α-α-corners, α-α-hairpins, and L- and V-shaped supersecondary structural elements on the surface of protein molecule. Introduction of PTM moiety affects meaningfully on distances between helices, torsion angle, length, area, and polygonal perimeter of helices within accounted motifs.

The assessment of protein structure geometry demonstrated, that modifiable amino acid residues are always exhibited on the surface accessible to surrounding solvent, and PTM increases the area of solvent-accessible surface for the immediate environment of amino acids (we designated this phenomenon as “active environment”). However, the total surface area of the exposed active environment can be even less than that of the unmodified polypeptide chain, suggesting that adjacent amino acid residues can reduce the surface area gain.

So far, the majority of examined PTMs characterize the phenomenon of "breathing of the molecule" and do not induce dramatic conformational changes following to structure instability. Conformational analysis of PTM-carrying supersecondary motifs permitted to imagine of protein surface and to assess contribution of PTMs in conformational fluctuations that possibly can alter protein activity.

The elaborated approach can drive a PTM-centric proteomics to evaluate the influence of PTM-caused structural flexibility, and encourage the discovery of currently underestimated PTM-encoded biomarkers for various diseases.

## Methods

### Demography and ethical consideration

The study population comprised of the group of patients with stage II–III breast cancer (*n* = 24, aged 48 ± 11 years old) and the group of patients with ovarian cancer at stages II-III (*n* = 53, aged 52 ± 12 years old), who had been inpatients at the M.F. Vladimirsky Research Clinics (Moscow). The control group comprised of 30 healthy volunteers (aged 36 ± 11 years old) with no previous oncological disease history. All groups under consideration were sex-aligned (only women participated in the study). Patients and healthy volunteers provided written Informed consent to participate in the study, and the study was approved by the local ethical committee of M.F. Vladimirsky Research Clinics (protocol no. 18 of December 24, 2020) and Sechenov University (protocol no 10–19 July 17, 2019) according to the WMA Declaration of Helsinki for ethical principles for medical research involving human subjects.

### Sample preparation for MS analysis

Following overnight fasting, peripheral blood samples (up to 5 mL) were collected in pre-chilled EDTA-2K^+^ vacuum tubes between 9 and 11 a.m. The collected blood samples were centrifuged for 10 min at 1500×*g* and t = 4 °C. The obtained plasma (supernatant) fraction was carefully transferred into clean cryotubes with a 2 mL nominal volume.

Blood plasma (4 µL) was mixed with a denaturation solution (1% deoxycholic acid sodium salt, 5 M urea, 6% acetonitrile, 300 mM sodium chloride, and buffered by 75 mM triethylammonium bicarbonate, pH 8.5) containing 10 mM TCEP (tris-(2-carboxyethyl) phosphine) up to 40 µL of final volume. The reaction mixture was incubated for 20 min at 45 °C under continual stirring. Then, 4 µL of 2% solution of 4-vinylpyridine in 30% isopropanol was added and the alkylating reaction was incubated for 20 min at ambient temperature in the dark place. Triethylammonium bicarbonate 50 mM (pH 8.2) was added to the sample to 500 µL final to make the concentration of urea and deoxycholic acid sodium salt safely for enzymatic digestion. Digestion was performed with trypsin (200 ng/µL supplemented in 30 mM acetic acid) in two stages: at the first stage, trypsin was added at a ratio of 1:50 (*w/w*) and the reaction was incubated for 3 h at 37 °C. In the second stage, the enzyme was added at a ratio of 1:100 (*w/w*), and the reaction was incubated at 37 °C for 12 h. After digestion completed, 25 µL of 50% formic acid was added to sediment deoxycholic acid sodium salt; samples were centrifuged at 12,500×*g* for 10 min at 10 °C, and obtained supernatant was dried under vacuum at 30 °C for 90–120 min. The resulting pellets were reconstituted in 40 µL of 0.5% formic acid.

### Survey mass spectrometry analysis

The analysis was conducted on a high-resolution Q Exactive-HF mass spectrometer (Thermo Scientific, Waltham, MA, USA) with an installed introduced nano-spray ionization (NSI) ionization source (Thermo Scientific). The selection of mass spectrometry parameters for data acquisition was the requirements of the Human Proteome Organization (HUPO Guidelines, bullet point 9, version 3.0.0, released October 15, 2019) for the minimal length of the detected peptide for consideration and justification of PE1 proteins (according to the Uniprot KB Classification).

Data acquisition was performed in a positive ionization mode in the range of 420–1250 m*/z* for precursor ions (with resolution *R* = 60 K) and in a range with the first recorded mass of 110 m*/z* for fragment ions (with resolution of *R* = 15 K). Precursor ions were accumulated for a maximum integration time of 15 ms, and fragment ions were accumulated for a maximum integration time of 85 ms. Top 20 precursor ions with a charge state between *z* = 2 + and *z* = 4 + were collected in the ion trap and pushed to the collision cell for fragmentation in high-energy collision dissociation mode. The activation energy was normalized at 27% for *m/z* = 524, *z* = 2 +, and ramped within ± 20% of the installed value.

Analytical separation was performed using an Ultimate 3000 RSLC Nano UPLC system (Thermo Scientific). Samples were quantitatively (2 μg) loaded onto the enrichment column Acclaim Pepmap® (5 × 0.3 mm, 300 Å pore size, 5 µm particle size) and washed at a flow rate of 20 μL/min for 4 min using a loading solvent (2.5% acetonitrile, 0.1% formic acid, and 0.03% acetic acid). Following the loading stage, peptides were separated on an Acclaim Pepmap® analytical column (75 µm × 150 mm, 1.8 µm particle size, 60 Å pore size) in a linear gradient of mobile phases A (water with 0.1% formic acid and 0.03% acetic acid) and B (acetonitrile with 0.1% formic acid and 0.03% acetic acid) at a flow rate of 0.3 μL/min. The following elution scheme was applied: the gradient started at 2.5% of B for 3 min and raised to 12% of B for the next 15 min, then to 37% of B for the next 27 min, and to 50% for the next 3 min. The gradient was rapidly increased to 90% of B for 2 min and was maintained for 8 min at a flow rate of 0.45 μL/min. Enrichment and analytical columns were equilibrated in the initial gradient conditions for the next 13 min at a flow rate of 0.3 μL/min before the following sample run. Mass spectrometric measurements were performed using the equipment of “Human Proteome” Core Facility (IBMC, Moscow, Russia).

### Targeted mass spectrometry analysis

Targeted mass spectrometry detection was performed on a high-resolution time-of-flight mass spectrometer Xevo G2-XS (Waters, the UK) equipped with a Z-spray ion source. The instrument operated in a positive electrostatic ionization mode with capillary voltage adjusted to 3 kV and cone voltage adjusted to 78 V with offset to 115 V. The desolvation gas flow rate was set to 680 L/h with a temperature of 350 °C, and the cone gas flow was set to 50 L/h with a temperature of 150 °C. Ions were acquired in a sensitive analyzer mode with a target mass enhancement. Fragment ions were acquired over the range of 150–1700 m*/z* with a fixed target mass set. Ions were surveys for 125 ms of duty cycle and decomposed at a collision energy ramped between 12 and 38 eV (the collision gas is argon) in CID (collision-induced dissociation) mode. Active on-line calibration using the Lock-mass *m/z* = 556.27 (Leu-Enkephalin, 50 pg/mL in 50% acetonitrile with 0.1% formic acid at a flow rate of 5 µL/min) within 5 mDa isolation window was applied every 45 s.

The acquisition method was designed in a segmental mode, where each segment corresponds to certain precursor ion detected within an estimated retention time window of ± 1.5 min. Precursor ions m/z were calculated using the MassLynx (version 4.2 SCN 996, Waters, the UK) mass calculator tool.

Peptides were loaded onto an Acquity™ UPLC BEHC18 (2.1 × 50 mm, 1.7 µm particle size; Waters, the UK) column heated to 40 °C and separated using an Acquity H Class UPLC system at a flow rate of 0.2 mL/min in a gradient of mobile phase A (water) and mobile phase B (acetonitrile) both supplied with 0.1% formic acid and 0.03% acetic acid by applying the following scheme of gradient: 0–1.5 min 3% of B, the raising the B to 19% at 26.5 min, then raising the B to 35% at 42 min and rapid increasing of B to 97% at 43.5 min, then keeping the isocratic mode of B for the next 4 min to 47.5 min at 0.3 mL/min flow rate, and moderate decrease to 3% (initial gradient condition) at 49 min. Column equilibration was lasted for the next 5 min at 3% of B at 0.2 mL/min.

### Protein identification and criteria selection for post-translational modifications

Adapted peak lists were searched using the OMSSA (version 2.1.9, Proteomics Resource, Seattle, WA, USA) search engine against a concatenated target/decoy protein sequence database UniProtKB (88703 (target) sequences with a restricted taxonomy (Homo sapiens). The decoy sequences were populated by the reverse sequence algorithm of the SearchGUI engine (release 3.1.16, Compomics, Gent-Zwijnaarde, Belgium).

Peptides were parsed with mass tolerance of 10.0 ppm for the MS1 (precursor) level and with a tolerance of 0.01 Da for the MS2 (fragment ions) level. Trypsin was set as a specific protease, and a maximum of two missed (internal) cleavages were allowed. Modifications of acetyl (acK), phospho (pS), phospho (pT), phospho (pY), and Gly-Gly (K) were selected as flexible. Peptides and proteins were identified using PeptideShaker search engine (version 2.0.22; Compomics, Gent-Zwijnaarde, Belgium) and validated at a 1.0% false discovery rate estimated as a decoy hit distribution.

To eliminate probable false positive results due to the concatenated search of several PTMs, we curated only those results that fit the following requirements: (a) at least 98% confidence for peptide identification, (b) at least 80% of peptide sequence coverage by fragmentation spectra, and (c) at least 10 units of D-score for PTM probability. Furthermore, the extracted data were manually curated to avoid possible false identifications. During this step, we removed those spectra that do not contain proper y/b fragment ion pairs designation that mark and locate the exact amino acid residue carrying PTM.

To consider the detectable PTM moieties as relevant to cancer phenotype and for the structural and molecular dynamic analysis, they should meet the following criteria: (a) the total set of PTM moieties must be detectable and identified in at least 50% of each cancer phenotype only and should not met in the control group; (b) each PTM moiety should be identified in at least 20% of PTM-carrying subjects.

### Analysis of post-translational protein modifications

In the present study, proteins containing tryptic peptides of a certain type of modification were selected from the PDB^53^. The selected proteins belong to the class of alpha-helical and globular proteins. For each peptide, a sample was created to determine the conformational template. Furthermore, all motifs containing the tryptic peptides were selected from the database. Our previously developed method was used to recognize and select structural motifs^54–56^.

The secondary structure of proteins was determined using the Kabsch and Sander DSSP methods^57^. Using the same program, the available contact surfaces with the solvent were determined. Definitions of important structural motif characteristics have been described in previous studies^54–56^. Visual analysis of the structures was performed using the RasMol molecular graphics program^58^.

In total, the DSSP program distinguishes three types of helices: α-helix, π-helix, and helix 3/10. The DSSP program also solves the problem of determining the beginning and end of the helix. A candidate for the desired structure (helical pair) is a protein region that contains two helices and a region of connection between helices. For each helix of the structure, the axis of the cylinder on which this helix was wound was determined using the least square method. The axis of the cylinder will be found more precisely when the closer the helix is to the ideal. The quality of the axis assessment was characterized by the value of the standard deviation. We selected those helices (and, accordingly, structures) for which the accuracy of the axis estimation satisfies a predetermined criterion. The two axes of the helices define the spatial structure completely. It is known that two parallel planes can be drawn through two non-intersecting straight lines in space such that the first axis belongs to the first plane and the second to the second plane. An axis lying in one plane can be projected onto another plane. Thus, the spatial structure is fully described by the distance between the parallel planes and the projections of the axes of the helices onto the plane.

To establish the stability of structural motifs without and considering the modification, a numerical experiment of molecular dynamics was carried out. Molecular dynamic simulation experiments were performed using the AMBER software (version 11)^59^. To calculate the molecular trajectories for the selected proteins, the experiments performed with no consideration of the water environment and at a field strength of AMBER ff03 at a temperature of 300 K^60^. Therefore, the complete energy of the considering system was minimized at the fixed atomic coordinates, which deployed the condition to organize the atomic interaction order. The molecular system was further heated up to the selected temperature (300 K), and molecular trajectories were recorded during 0.5 ns every 0.005 ns. The resulting molecular trajectories were visualized using the VMD (version 1.9.1) software^61^. The free energy for the binding profile of the molecular complexes was estimated by Born’s method^62^, and the distance between atoms was calculated using CPPTRAJ software (a part of AMBER (version 11)). The spatial geometry of polypeptide chains was characterized according to the categorized rules of Kabsch and Sander^57^.

The following geometric features^17^ of the assayed motifs with mounted PTMs were recorded every 0.005 ns: minimal (r) and inter-planar (d) distances between helices, α- and torsion (θ) angles between axes of helices, area (S), and perimeter (P) of polygons of the helices projections intersection. Means and standard deviations were recorded and utilized to defined the stability of protein molecule relatively to the initial intact condition. The geometric features were recorded for motifs that met the following criteria: (1) the presence of a PTM moiety, (2) the PTM must be localized on or close to the helix or at least on the polypeptide chain lining the affected helices; (3) helices should be in tight contact and the minimal and inter-planar distances must be equal (r = d ≤ 16 Å), but the area and the perimeter should have a non-null and not close to zero^54–57^.

### Statistical analysis

Principal component analysis (PCA) was performed for the set of proteins shared between the groups of study. To be selected from the total proteome, the candidate protein should meet the criterion of unicity among the totality identified peptides. To assess the similarity of identified proteomes an upset plot was generated using the UpSetR function in UpSetR^63^.

The null values of intensity were replaced to the mass spectrometric instrumental-adjusted threshold equal to105 counts. Protein intensity was estimated as the normalized summed peptide intensities belonged to the certain protein. The resulting matrix of protein intensities was used for the quantification based on the calculation of intensity medians for each protein within studied groups. Wilcoxon test with a threshold of *p* < 0.05 was applied to sampled groups of study. Measure of protein abundancy was represented as a median value fold changes (FC) ratio toward the control group. Proteins attributed to certain pathology groups and featured by log (2)-fold changes (log_2_(FC)) cut-off more than 1 or less than − 1, and a significance *p*-values of < 0.05 (Wilcoxon test) were considered as meaningfully different in quantitative property. Frequency was estimated for protein identifications with the intensity exceeded the adjusted instrumental threshold of 105 counts. Significantly altered proteins were submitted for functional and pathways annotation analysis at a q-value threshold less than *q* < 0.01 using PANTHER Overrepresentation Test of Gene Ontology toolset^64^, and Bonferroni correction for multiple testing has been applied. The enriched terms were refined with similarity coefficient of > 0.7.

## Supplementary Information

Supplementary Information.
