# Supplementary material for: Proteomic and molecular dynamic investigations of PTM-induced structural fluctuations in breast and ovarian cancer
Source: Sci Rep. 2021 Sep 29;11:19318. doi: 10.1038/s41598-021-98201-7 (PMC8481388; doi:10.1038/s41598-021-98201-7)
Supplement: Supplementary file 1 — Supplementary Information. [file 41598_2021_98201_MOESM1_ESM.pdf]

Structural changes in proteins with post-translational modifications in female oncopathologies

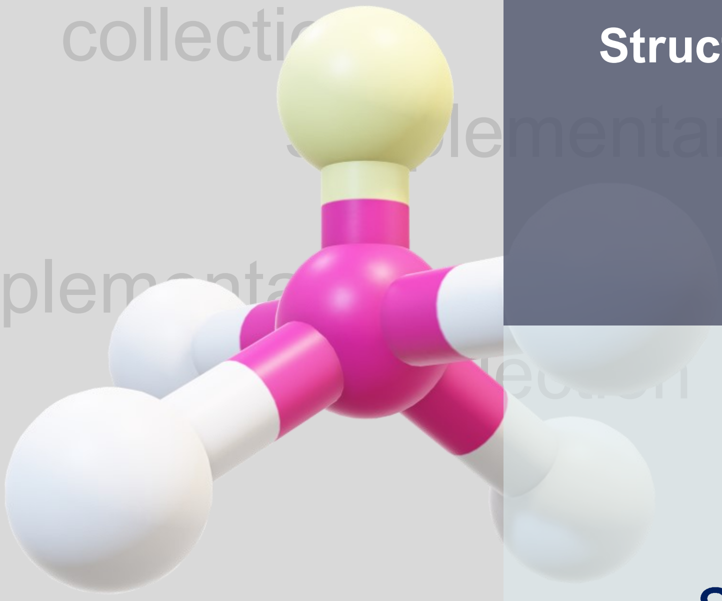

supplementary materials collection

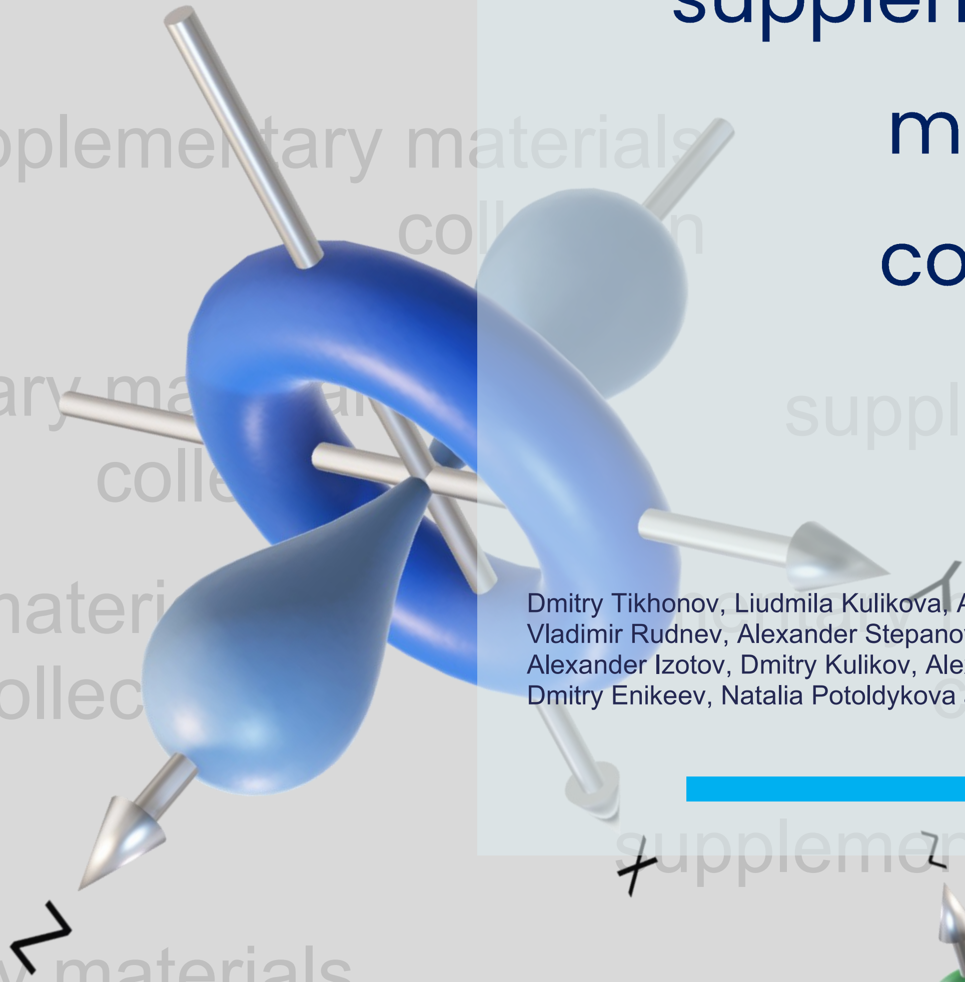

Dmitry Tikhonov, Liudmila Kulikova, Arthur Kopylov, Vladimir Rudnev, Alexander Stepanov Kristina Malsagova, Alexander Izotov, Dmitry Kulikov, Alexey Zulkarnaev, Dmitry Enikeev, Natalia Potoldykova and Anna Kaysheva

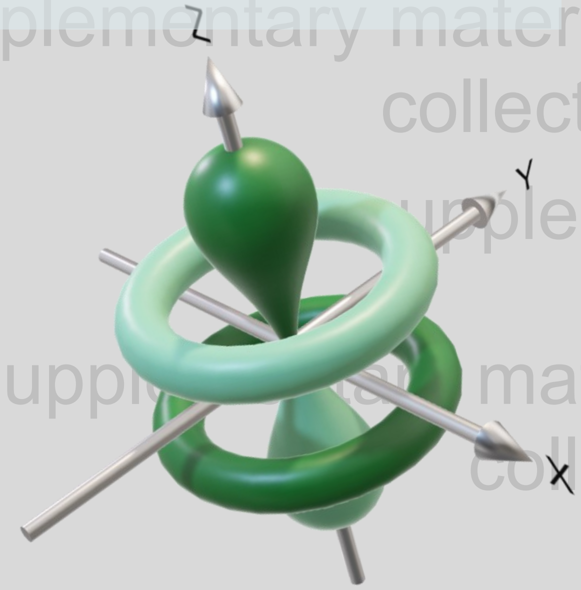

The defined proteome specified for group of patients with ovarian cancer or patients with breast cancer

Comparative symmetry analysis showed that the mutual proteome size (between all studied groups) was *n* = 147 protein identifications. The common proteome size of two cancer-specific groups was n = 50 protein identifications, whereas the group-specific proteomes were *n* = 74 and *n* = 25 protein identifications amongst patients with ovarian and breast cancer, correspondingly. Proteins, attributed for the pathology groups and featured by a statistically significant between-groups quantitative difference, are given below in **Supplementary Table S1**.

Supplementary Table S1: Plasma-based differentially expressed proteins (DEP) selected upon results of semi-quantitative and symmetry analysis at statistical significance cut-off level of $p<0.05$.

OVARIAN CANCER							
**Gene**	**Protein name**	**p-value**	**log10 p-value**	**Frequency in OC**	**frequency in control**	**log2 FC**	**Biological Process (Gene Ontology)**
PZP	Pregnancy zone protein	1.90E-04	3.72	0.77	0.18	9.86	GO:0030162 regulation of proteolysis
SHBG	Sex hormone-binding globulin	1.90E-04	3.72	0.79	0.27	7.92	n/d
CPN2	Carboxypeptidase N subunit 2	9.10E-05	4.04	0.94	0.36	7.24	GO:0030162 regulation of proteolysis
SERPINA7	Thyroxine-binding globulin	1.28E-06	5.89	0.94	0.09	6.72	GO:0030162 regulation of proteolysis
F13B	Coagulation factor XIII B chain	2.89E-03	2.54	0.74	0.27	5.61	GO:0072376 protein activation cascade
SAA1	Serum amyloid A-1 protein	4.68E-02	1.33	0.57	0.18	4.43	GO:0051246 regulation of protein metabolic process
CNDP1	Beta-Ala-His dipeptidase	1.02E-02	1.99	0.58	0.09	3.81	GO:0051246 regulation of protein metabolic process
AGT	Angiotensinogen	2.11E-05	4.68	1	1	1.75	GO:0030162 regulation of proteolysis
C5	Complement C5	3.72E-03	2.43	0.94	1	1.12	GO:0072376 protein activation cascade
C3	Complement C3	2.00E-04	3.7	1	1	-1.07	GO:0072376 protein activation cascade
VTN	Vitronectin	1.05E-04	3.98	1	1	-1.12	GO:0030162 regulation of proteolysis
C1S	Complement C1s subcomponent	2.38E-02	1.62	0.94	1	-1.17	GO:0072376 protein activation cascade
APOB	Apolipoprotein B-100	9.33E-04	3.03	1	1	-1.19	GO:0002682 regulation of immune system process
CFB	Complement factor B	1.06E-03	2.97	0.98	1	-1.22	GO:0072376 protein activation cascade
ITIH2	Inter-alpha-trypsin inhibitor heavy chain H2	5.50E-03	2.26	1	1	-1.25	GO:0030162 regulation of proteolysis
AMBP	Protein AMBP	1.03E-03	2.99	1	1	-1.32	GO:0030162 regulation of proteolysis
PON1	Serum paraoxonase/arylesterase 1	4.24E-02	1.37	0.91	1	-1.37	n/d
SERPINA3	Alpha-1-antichymotrypsin	8.76E-04	3.06	1	1	-1.39	n/d
APOE	Apolipoprotein E	2.14E-04	3.67	1	1	-1.45	GO:0030162 regulation of proteolysis
MLXIPL	Carbohydrate-responsive element-binding protein	8.50E-04	3.07	0.7	1	-1.5	GO:0051246 regulation of protein metabolic process
CLEC3B	Tetranectin	4.79E-06	5.32	0.92	1	-1.65	GO:0030162 regulation of proteolysis
LRG1	Leucine-rich alpha-2-glycoprotein	5.20E-03	2.28	0.91	1	-1.68	GO:0006810 transport
C9	Complement component C9	3.63E-05	4.44	0.89	1	-1.69	GO:0072376 protein activation cascade
C8G	Complement component C8 gamma chain	2.66E-03	2.57	0.72	0.91	-1.78	GO:0072376 protein activation cascade
KNG1	Kininogen-1	6.19E-06	5.21	0.98	1	-1.8	GO:0072376 protein activation cascade
HPX	Hemopexin	9.04E-07	6.04	1	1	-1.85	GO:0051246 regulation of protein metabolic process
APOA1	Apolipoprotein A-I	1.19E-06	5.93	1	1	-1.88	GO:0051246 regulation of protein metabolic process
CLU	Clusterin	3.13E-06	5.51	0.96	1	-1.94	GO:0072376 protein activation cascade
HBB	Hemoglobin subunit beta	9.37E-03	2.03	1	1	-1.95	GO:0019222 regulation of metabolic process
HP	Haptoglobin	9.02E-06	5.04	0.81	1	-1.98	GO:0006952 defense response
ORM1	Alpha-1-acid glycoprotein 1	1.74E-04	3.76	1	1	-2.04	GO:0006952 defense response
APOA2	Apolipoprotein A-II	6.88E-07	6.16	1	1	-2.05	GO:0051246 regulation of protein metabolic process
SAA4	Serum amyloid A-4 protein	2.00E-06	5.7	0.89	1	-2.1	GO:0006952 defense response
APOD	Apolipoprotein D	6.24E-07	6.21	0.91	1	-2.2	GO:0051246 regulation of protein metabolic process
F2	Prothrombin	1.19E-06	5.93	1	1	-2.21	GO:0072376 protein activation cascade
SERPINF2	Alpha-2-antiplasmin	7.31E-06	5.14	1	1	-2.26	GO:0030162 regulation of proteolysis
RBP4	Retinol-binding protein 4	2.89E-05	4.54	0.96	1	-2.29	GO:0002682 regulation of immune system process
PROS1	Vitamin K-dependent protein S	1.57E-05	4.8	0.66	1	-2.43	GO:0030162 regulation of proteolysis
APOC1	Apolipoprotein C-I	3.13E-06	5.5	1	1	-2.44	GO:0019222 regulation of metabolic process
APOA4	Apolipoprotein A-IV	2.21E-06	5.66	0.98	1	-2.49	GO:0006952 defense response
SERPINA4	Kallistatin	4.16E-02	1.38	0.81	0.73	-2.6	GO:0030162 regulation of proteolysis

APCS	Serum amyloid P-component	1.91E-05	4.72	0.6	1	-2.65	GO:0051246 regulation of protein metabolic process
C1R	Complement C1r subcomponent	4.55E-04	3.34	0.94	1	-2.68	GO:0072376 protein activation cascade
APOC2	Apolipoprotein C-II	6.86E-07	6.16	0.92	1	-2.78	GO:0019222 regulation of metabolic process
AZGP1	Zinc-alpha-2-glycoprotein	1.86E-04	3.73	0.96	1	-2.86	GO:0006810 transport
APOH	Beta-2-glycoprotein 1	3.13E-06	5.5	1	1	-3.13	GO:0072376 protein activation cascade
APOM	Apolipoprotein M	6.83E-06	5.17	0.66	1	-3.25	GO:0006810 transport
GSN	Gelsolin	2.71E-07	6.57	0.98	1	-3.27	GO:0030162 regulation of proteolysis
SERPING1	Plasma protease C1 inhibitor	2.24E-07	6.65	0.98	1	-3.28	GO:0072376 protein activation cascade
ORM2	Alpha-1-acid glycoprotein 2	2.24E-07	6.65	0.96	1	-3.41	GO:0006952 defense response
C2	Complement C2	4.53E-03	2.34	0.81	0.82	-3.41	GO:0072376 protein activation cascade
SERPINC1	Antithrombin-III	2.24E-07	6.65	0.98	1	-3.65	GO:0030162 regulation of proteolysis
HRG	Histidine-rich glycoprotein	2.43E-07	6.61	0.89	1	-3.7	GO:0072376 protein activation cascade
FN1	Fibronectin	4.72E-07	6.33	0.91	1	-3.76	GO:0030162 regulation of proteolysis
KLKB1	Plasma kallikrein	4.05E-07	6.39	0.79	1	-4.63	GO:0072376 protein activation cascade
LPA	inter-alpha-trypsin inhibitor heavy chain H2	1.30E-03	2.89	0.23	0.64	-5.28	GO:0030162 regulation of proteolysis
CFHR1	Complement factor H-related protein 1	1.77E-04	3.75	0.11	0.55	-6.18	GO:0072376 protein activation cascade
APOF	Apolipoprotein F	7.23E-09	8.14	0.26	1	-7.09	GO:0006810 transport
EFCAB7	EF-hand calcium-binding domain-containing protein 7	2.50E-11	10.6	0.02	0.82	-8.54	GO:0019222 regulation of metabolic process
C4A	Complement C4-A	1.87E-07	6.73	0.72	1	-8.81	GO:0072376 protein activation cascade
FGA	Fibrinogen alpha chain	2.04E-07	6.69	0.77	1	-9.76	GO:0072376 protein activation cascade
FGG	Fibrinogen gamma chain	1.12E-08	7.95	0.32	1	-14.86	GO:0072376 protein activation cascade
FGB	Fibrinogen beta chain	5.52E-10	9.26	0.19	1	-15.12	GO:0072376 protein activation cascade
PZP	Pregnancy zone protein	1.90E-04	3.72	0.77	0.18	9.86	GO:0030162 regulation of proteolysis
SHBG	Sex hormone-binding globulin	1.90E-04	3.72	0.79	0.27	7.92	n/d
**BREAST CANCER**							
**Gene**	**Protein name**	**p-value**	**log10 p-value**	**Frequency in OC**	**frequency in control**	**log2 FC**	**Biological Process (Gene Ontology)**
TTR	Transthyretin	4.05E-02	1.39	0.92	0.64	1.55	GO:0006887 exocytosis
APOE	Apolipoprotein E	1.36E-03	2.87	0.96	1	-1.34	GO:0030162 regulation of proteolysis
C1QB	Complement C1q subcomponent subunit B	2.82E-02	1.55	0.58	0.82	-1.73	GO:0030162 regulation of proteolysis
APOC3	Apolipoprotein C-III	3.36E-08	7.47	1	1	-2.72	n/d
VTN	Vitronectin	1.92E-08	7.72	1	1	-2.74	GO:0030162 regulation of proteolysis
TF	Serotransferrin	2.28E-04	3.64	1	1	-2.85	GO:0051346 negative regulation of hydrolase activity
AMBP	Protein AMBP	2.16E-07	6.67	0.96	1	-2.97	GO:0030162 regulation of proteolysis
GC	Vitamin D-binding protein	5.36E-04	3.27	1	1	-3.05	n/d
ITIH4	Inter-alpha-trypsin inhibitor heavy chain H4	8.92E-04	3.05	0.92	1	-3.06	GO:0030162 regulation of proteolysis
APOC1	Apolipoprotein C-I	1.34E-05	4.87	0.88	1	-3.07	GO:0019222 regulation of metabolic process
C7	Complement component C7	1.09E-03	2.96	0.58	0.91	-3.16	GO:0006957 complement activation, alternative pathway
SERPINA1	Alpha-1-antitrypsin	1.77E-06	5.75	1	1	-3.17	n/d
APOA2	Apolipoprotein A-II	9.35E-07	6.03	1	1	-3.18	GO:0051246 regulation of protein metabolic process
C1QC	Complement C1q subcomponent subunit C	1.51E-03	2.82	0.5	1	-3.28	GO:0006958 complement activation, classical pathway
SAA4	Serum amyloid A-4 protein	3.29E-05	4.48	0.58	1	-3.52	GO:0006952 defense response
A1BG	Alpha-1B-glycoprotein	1.30E-06	5.89	1	1	-3.52	GO:0006887 exocytosis
ITIH2	Inter-alpha-trypsin inhibitor heavy chain H2	7.23E-05	4.14	1	1	-3.6	GO:0030162 regulation of proteolysis
PLG	Plasminogen	1.58E-05	4.8	0.92	1	-3.62	GO:0006887 exocytosis
CP	Ceruloplasmin	6.92E-06	5.16	0.88	1	-3.62	n/d
RBP4	Retinol-binding protein 4	3.96E-05	4.4	0.79	1	-3.65	GO:0002682 regulation of immune system process
ITIH1	Inter-alpha-trypsin inhibitor heavy chain H1	2.47E-05	4.61	1	1	-3.65	n/d
AFM	Afamin	5.29E-04	3.28	0.88	1	-3.76	n/d
C5	Complement C5	2.29E-03	2.64	0.54	1	-3.85	GO:0072376 protein activation cascade
APOA1	Apolipoprotein A-I	4.79E-09	8.32	1	1	-3.87	GO:0051246 regulation of protein metabolic process
PGLYRP2	N-acetylmuramoyl-L-alanine amidase	4.41E-05	4.36	0.71	1	-3.99	n/d
AZGP1	Zinc-alpha-2-glycoprotein	2.61E-03	2.58	0.75	1	-4.07	GO:0006810 transport
APOD	Apolipoprotein D	2.60E-06	5.59	0.67	1	-4.07	GO:0051246 regulation of protein metabolic process
C3	Complement C3	2.16E-07	6.67	1	1	-4.07	GO:0072376 protein activation cascade
KNG1	Kininogen-1	3.42E-06	5.47	0.79	1	-4.17	GO:0072376 protein activation cascade
GSN	Gelsolin	3.42E-06	5.47	0.79	1	-4.19	GO:0030162 regulation of proteolysis
APOC2	Apolipoprotein C-II	4.19E-06	5.38	0.54	1	-4.29	GO:0019222 regulation of metabolic process

LBP	Lipopolysaccharide-binding protein	1.01E-04	4	0.04	0.64	-4.44	GO:0051246 regulation of protein metabolic process
APOA4	Apolipoprotein A-IV	1.72E-05	4.76	0.71	1	-4.46	GO:0006952 defense response
C8B	Complement component C8 beta chain	4.71E-02	1.33	0.21	0.55	-4.62	GO:0006957 complement activation, alternative pathway
C4B	Complement C4-B	1.40E-04	3.85	0.54	1	-4.66	GO:0006958 complement activation, classical pathway
CFH	Complement factor H	2.16E-05	4.67	0.88	1	-4.68	GO:0006957 complement activation, alternative pathway
APOB	Apolipoprotein B-100	7.98E-06	5.1	0.79	1	-4.71	GO:0002682 regulation of immune system process
SERPINC1	Antithrombin-III	2.95E-06	5.53	0.88	1	-4.72	GO:0030162 regulation of proteolysis
APCS	Serum amyloid P-component	1.85E-06	5.73	0.5	1	-4.74	GO:0051246 regulation of protein metabolic process
C4A	Complement C4-A	9.59E-09	8.02	1	1	-4.75	GO:0072376 protein activation cascade
APOM	Apolipoprotein M	8.88E-06	5.05	0.5	1	-4.89	GO:0006810 transport
CFB	Complement factor B	1.54E-05	4.81	0.79	1	-5.04	GO:0072376 protein activation cascade
SERPINA3	Alpha-1-antichymotrypsin	6.95E-06	5.16	0.92	1	-5.04	n/d
F12	Coagulation factor XII	9.05E-04	3.04	0.5	1	-5.04	GO:0072376 protein activation cascade
SERPINF2	Alpha-2-antiplasmin	9.60E-05	4.02	0.75	1	-5.23	GO:0030162 regulation of proteolysis
FCN3	Ficolin-3	1.56E-03	2.81	0.08	0.55	-5.24	GO:0072376 protein activation cascade
HBB	Hemoglobin subunit beta	2.08E-03	2.68	1	1	-5.3	GO:0019222 regulation of metabolic process
F2	Prothrombin	2.97E-06	5.53	0.92	1	-5.32	GO:0072376 protein activation cascade
SERPIND1	Heparin cofactor 2	4.49E-05	4.35	0.58	1	-5.38	GO:0030162 regulation of proteolysis
ORM1	Alpha-1-acid glycoprotein 1	9.59E-09	8.02	1	1	-5.59	GO:0006952 defense response
FN1	Fibronectin	5.71E-06	5.24	0.79	1	-5.65	GO:0030162 regulation of proteolysis
HPX	Hemopexin	4.79E-09	8.32	0.96	1	-5.7	GO:0051246 regulation of protein metabolic process
CLU	Clusterin	9.42E-06	5.03	0.79	1	-5.79	GO:0072376 protein activation cascade
SERPING1	Plasma protease C1 inhibitor	2.72E-06	5.57	0.71	1	-6.02	GO:0072376 protein activation cascade
APOL1	Apolipoprotein L1	1.68E-03	2.77	0.13	0.64	-6.32	n/d
APOH	Beta-2-glycoprotein 1 GPI)	5.85E-06	5.23	0.88	1	-6.33	GO:0072376 protein activation cascade
C8G	Complement component C8 gamma chain	1.24E-06	5.91	0.08	0.91	-6.58	GO:0006957 complement activation, alternative pathway
C6	Complement component C6	5.99E-05	4.22	0.29	0.91	-6.64	GO:0030162 regulation of proteolysis
C1S	Complement C1s subcomponent	1.44E-04	3.84	0.42	1	-6.86	GO:0072376 protein activation cascade
CLEC3B	Tetranectin	1.61E-06	5.79	0.46	1	-6.89	GO:0030162 regulation of proteolysis
SERPINF1	Pigment epithelium-derived factor	7.89E-06	5.1	0.46	1	-6.96	GO:0030162 regulation of proteolysis
HPR	Haptoglobin-related protein	1.72E-04	3.76	0.13	0.82	-7.02	n/d
APOF	Apolipoprotein F	3.39E-08	7.47	0.04	1	-7.09	GO:0006810 transport
C8A	Complement component C8 alpha chain	7.67E-07	6.12	0.17	1	-7.37	GO:0006957 complement activation, alternative pathway
SERPINA6	Corticosteroid-binding globulin	1.19E-04	3.92	0.46	1	-7.5	GO:0030162 regulation of proteolysis
ORM2	Alpha-1-acid glycoprotein 2	2.45E-06	5.61	0.63	1	-7.59	GO:0006952 defense response
PROS1	Vitamin K-dependent protein S	1.32E-07	6.88	0.13	1	-7.73	GO:0030162 regulation of proteolysis
C2	Complement C2	1.15E-04	3.94	0.29	0.82	-7.91	GO:0072376 protein activation cascade
CFI	Complement factor I	1.88E-05	4.72	0.42	1	-8.18	GO:0006958 complement activation, classical pathway
IGFALS	Insulin-like growth factor-binding protein complex acid labile subunit	2.59E-06	5.59	0.29	1	-8.23	n/d
CD5L	CD5 antigen-like	5.26E-06	5.28	0.13	1	-8.26	n/d
MLXIPL	Carbohydrate-responsive element-binding protein	3.86E-07	6.41	0.13	1	-8.37	GO:0051246 regulation of protein metabolic process
STXBP5	Syntaxin-binding protein 5 giant larvae protein homolog 3)	1.66E-04	3.78	0.08	0.73	-8.54	GO:0006887 exocytosis
EFCAB7	EF-hand calcium-binding domain-containing protein 7	1.15E-05	4.94	0.13	0.82	-8.54	GO:0019222 regulation of metabolic process
C1R	Complement C1r subcomponent	3.39E-06	5.47	0.42	1	-8.66	GO:0072376 protein activation cascade
SERPINA4	Kallistatin	9.37E-04	3.03	0.29	0.73	-8.71	GO:0030162 regulation of proteolysis
LRG1	Leucine-rich alpha-2-glycoprotein	9.35E-06	5.03	0.46	1	-9.05	GO:0006810 transport
PON1	Serum paraoxonase/arylesterase 1	8.04E-06	5.09	0.33	1	-9.7	n/d
C4BPA	C4b-binding protein alpha chain	9.27E-06	5.03	0.13	1	-9.85	GO:0006958 complement activation, classical pathway
KLKB1	Plasma kallikrein	1.64E-07	6.79	0.13	1	-9.95	GO:0072376 protein activation cascade
FGA	Fibrinogen alpha chain	2.72E-06	5.57	0.71	1	-9.99	GO:0072376 protein activation cascade
C9	Complement component C9	1.09E-06	5.96	0.13	1	-10.11	GO:0072376 protein activation cascade
HRG	Histidine-rich glycoprotein	1.98E-06	5.7	0.42	1	-10.79	GO:0072376 protein activation cascade
HP	Haptoglobin	2.21E-06	5.65	0.5	1	-13.13	GO:0006952 defense response
FGG	Fibrinogen gamma chain	9.00E-07	6.05	0.33	1	-14.86	GO:0072376 protein activation cascade
FGB	Fibrinogen beta chain	6.87E-07	6.16	0.29	1	-15.12	GO:0072376 protein activation cascade
TTR	Transthyretin	4.05E-02	1.39	0.92	0.64	1.55	GO:0006887 exocytosis

We focused in on three known types of PTMs to identify them amongst patients under consideration (Figure S1). The discovered PTMs were ranged and plotted against each study group to reveal their input. It has been found, that ubiquitination is the least prevalent type of PTM in cancer phenotypes, but can be readily extracted from healthy donors. At the same time, phosphorylation was found to be most frequent type of PTM in ovarian cancer patients with the local input of 55%.

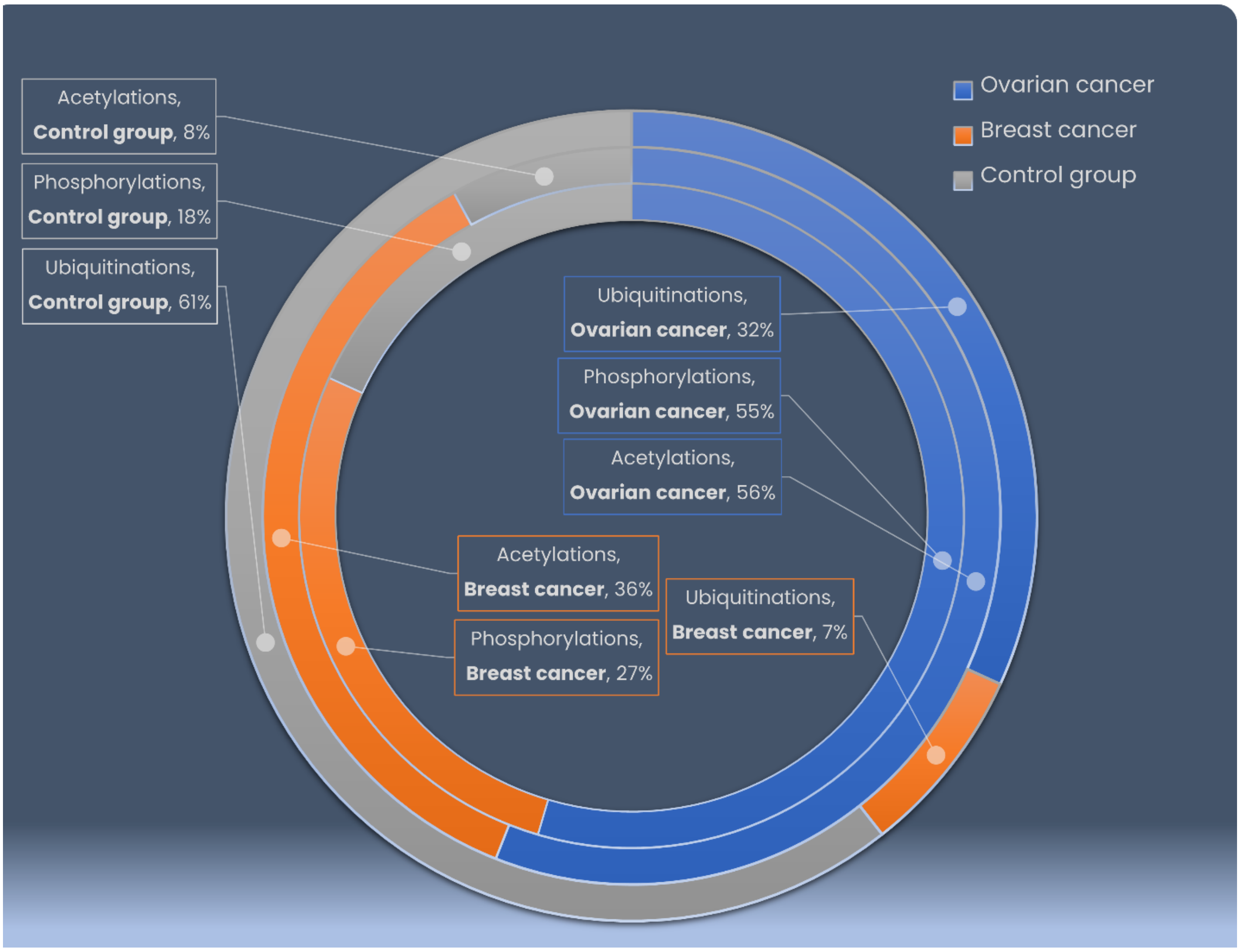

Supplementary Figure S1: Distribution of identified PTMs types within studied groups. The defined PTMs of interest are distributed unequally amigo different cancer groups and the control group. Particularly, ubiquitination of the most prevalent type of PTM among healthy donors in contrast to cancer phenotype patients, where this PTM made only 32% and 7% in ovarian and breast cancer, correspondingly. Likewise, acetylation is more general PTM for cancer phenotype and constitutes only 8% on the control group of healthy donors. Altogether, it may partially conform the known phenomenon of actively ongoing chromatin remodeling and significantly suppressed apoptosis processes in tumor cells.

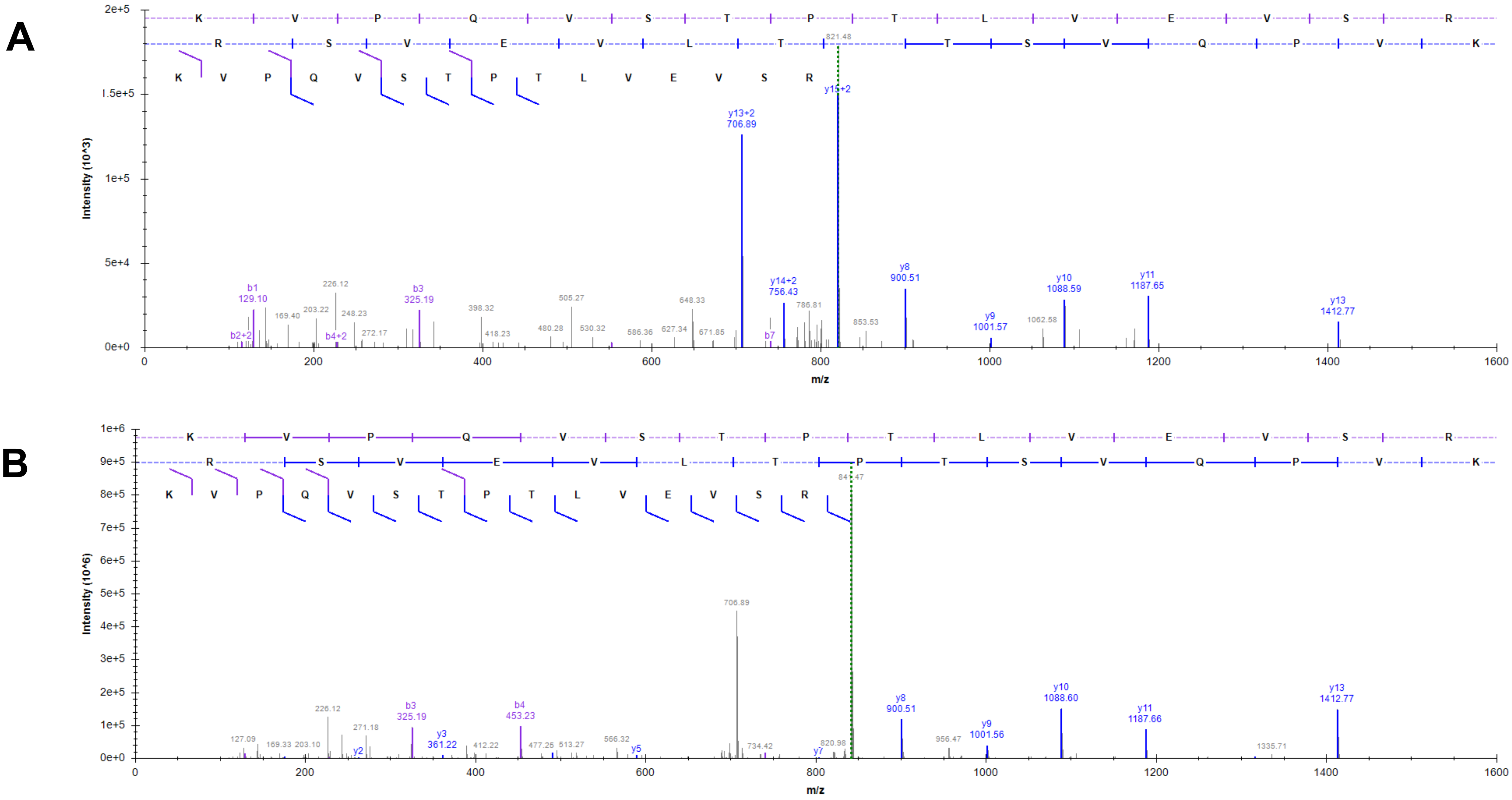
A
B
Intensity (10^3)
Intensity (10^6)
m/z
K V P Q V S T P T L V E V S R
R S V E V L T P T S V Q P V K
b1 129.10
b2+2
b3 325.19
b4+2
b7
y13+2 706.89
y15+2
821.48
y14+2 756.43
y8 900.51
y9 1001.57
y10 1088.59
y11 1187.65
y13 1412.77
b3 325.19
b4 453.23
y2
y3 361.22
y5
y7
706.89
y8 900.51
y9 1001.56
y10 1088.60
y11 1187.66
y13 1412.77

C

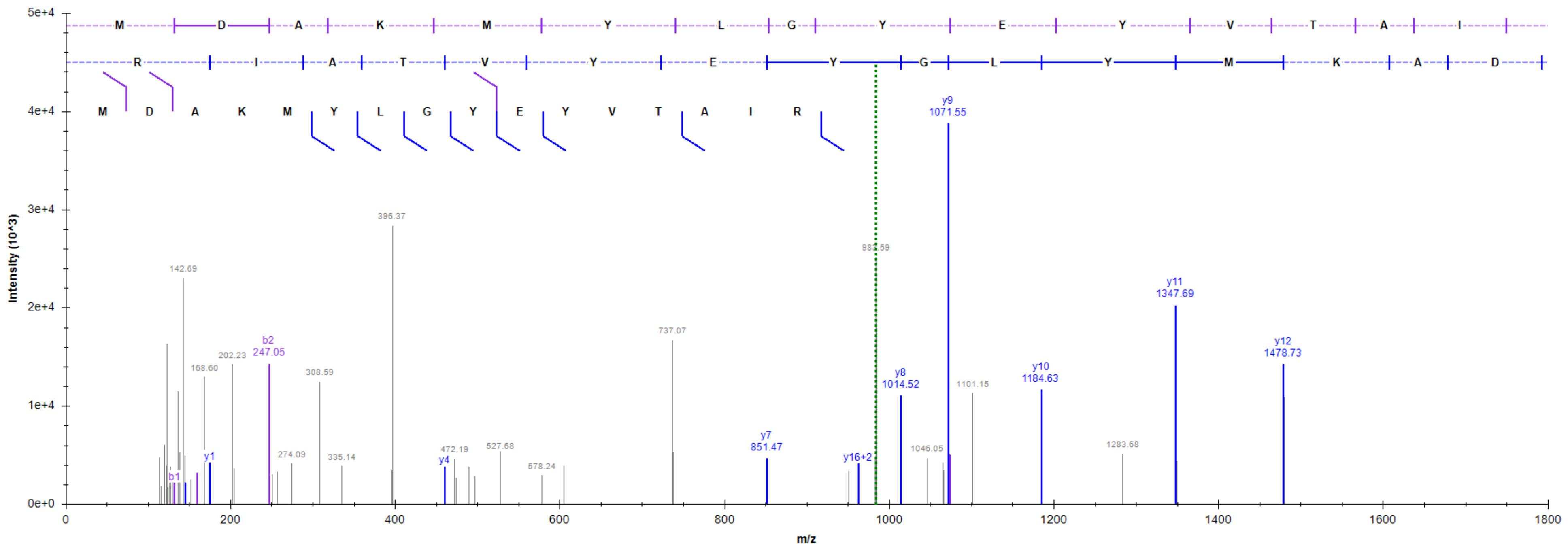

Supplementary Figure S2: Fragmentation spectra of the selected peptides obtained after targeted MS2 analysis. Top panel (**A**) shows fragmentation of intact(unmodified) peptide KVPQVSTPTLVEVSR (ALBU) detected as an ion with m/z m/z=820.4719^{2+} (mass error is -0.73 ppm) at 14.12 minutes (see Figure 2 in the Main text); middle panel (**B**) demonstrates the fragmentation spectrum of its modified counterpart acetylated at lysine, and detected as an ion with m/z=841.4774^{2+} (mass error is -0.83 ppm) at 14.46 minutes (see Figure 2 in the Main test). Bottom panel (**C**) shows the fragmentation spectrum of the modified peptide with amino acid sequence MDAKMYLGYEYVTAIR derived from TRFE and found in both ovarian and breast cancer groups of study. The peptide is acetylated at lysine and was detected as an ion with m/z=983.4725 (mass error is -0.91 pm) at 34.47 minutes.

There were five different PTMs identified specifically in serum samples of patients with breast cancer or ovarian cancer only and were not detected amongst patients of the control group. Each of selected PTMs were further categorized by the spatial geometry feature assisted by the molecular dynamic experiments.

The following characteristics were calculated along the molecular dynamic itemizing:

- LocPTM –type and localization of the modified amino acid residue in the chain-A;
- rA, rB – is a root mean square deviations (RMSD) for helices;
- d – mean value of inter-planar distance;
- r – minimal distance;
- α – angle between helices axis;
- θ – torsion angle;
- S – area of intercept between helices;
- P – polygonal perimeter between helices;
- sd, sr, sα, sθ, sS, sP – root mean square deviation of the above-mentioned parameters;
- *stat* – qualification pattern of the original (initial) structure;
- *nmd* – qualification of the unmodified (intact) structure;
- *mod* – qualification of the modified structure;

Supplementary Table S2: Molecular dynamics simulation results of the geometry calculations for modified albumin

Locus			Helixes between I^{st} and II^{d} helix	d (±SD), Å	r (±SD), Å	Θ (±SD), rad	S (±SD), Å^2	P (±SD), Å	Structure
PTM	I^{st} helix	II^{d} helix							
p(Y)-146	12–26	147–166	5	10,4±0	10,4±0	-69±0	97,2±0	40,8±0	stat
				10,3±0,9	10,4±0,8	-73±6	64,6±16,4	35,8±3	nmd
				11,9±1,6	12±1,6	-73±5	77±11,6	37,6±2,1	mod
	116–125	147–166	1	10,3±0	10,3±0	54±0	119,2±0	43,2±0	stat
				12,5±0,8	12,5±0,8	69±5	118±13,8	43,2±2,4	nmd
				12,4±1,2	12,4±1,2	70±7	107,8±17,9	41,3±3,4	mod
	127–141	147–166	no	10,4±0	10,4±0	-35±0	181,3±0	54,9±0	stat
				10,2±0,5	10,2±0,5	-38±3	169±10,7	53,5±0,9	nmd
				11,6±0,7	11,6±0,7	-35±3	177,7±7,6	54,4±1,1	mod
	147–166	279–287	4	9,6±0	9,6±0	68±0	87,1±0	37,5±0	stat

				10±1,3	10±1,3	81±9	67±19,9	33,4±4,6	nmd
				11,7±1,4	11,7±1,4	70±8	90,3±13	38,3±2,3	mod
ac(K)-319	224–243	319–333	4	11,2±0	11,2±0	-48±0	95±0	39,9±0	stat
				10,7±0,8	10,9±0,8	-43±3	56,1±18,5	33,2±3,3	nmd
				10,4±0,8	10,6±0,7	-42±3	52,7±16,8	32,8±2,9	mod
	**319–333**	**339–357**	no	10±0	10±0	-36±0	181,3±0	55±0	stat
				10,3±0,4	10,3±0,4	-33±2	184,3±4,1	55±0,5	nmd
				10,1±0,3	10,1±0,3	-36±3	179,4±5,7	54,7±0,5	mod
	319–333	368–394	1	15,2±0	15,2±0	127±0	114,7±0	42,2±0	stat
				16,7±0,7	16,7±0,7	132±4	127,5±13,5	43,8±2,3	nmd
				15,4±0,5	15,4±0,5	128±3	116,5±9,1	42,1±1,1	mod
ac(K)-374	319–333	368–394	1	15,2±0	15,2±0	127±0	114,7±0	42,2±0	stat
				16,7±0,7	16,7±,07	132±4	127,5±13,5	43,8±2,3	nmd
				15,6±0,6	15,7±0,6	135±5	94±22,1	41±2,7	mod
	**339–357**	**368–394**	no	9,5±0	9,5±0	-30±0	145,2±0	52,1±0	stat
				9,5±1,1	9,9±0,8	-32±3	104,6±29	48,2±3,3	nmd
				6,6±1,8	8±1	-45±10	66,5±29,3	39,7±6	mod
	339–357	416–434	1	9,3±0	9,3±0	52±0	115±0	43,3±0	stat
				8,7±0,5	8,7±0,5	54±4	96,7±10,9	39,8±2	nmd
				9,5±0,6	9,5±0,6	50±4	74,9±19,1	36,4±3,2	mod
ac(K)-410	**396–410**	**416–434**	no	8±0	8±0	-30±0	174,4±0	55,5±0	stat
				8,8±0,4	8,8±0,4	-32±2	172,7±5,5	55,1±1	nmd
				9,3±0,6	9,3±0,6	-33±2	172±6,6	54,7±1,1	mod
	396–410	440–462	1	15±0	15±0	104±0	151,5±0	52,1±0	stat
				16,5±0,5	16,5±0,5	111±4	137,4±14,8	50,4±2,3	nmd
				16,1±1,1	16,1±1,1	117±5	119,7±19,3	48,4±3	mod
ac(K)-541	396–410	538–555	5	11,7±0	11,7±0	-57±0	110,2±0	43±0	stat
				12,7±1	12,9±1	-85±16	54,3±31,9	32,3±6,1	nmd
				12,9±2,2	15,6±3,4	-109±28	22,9±35,8	12,1±16,4	mod
	**514–530**	**538–555**	no	10,6±0	10,6±0	-35±0	194,2±0	59,6±0	stat
				11,8±0,7	11,8±0,7	-40±4	171,8±15,6	55,5±2,6	nmd
				10,6±0,5	10,6±0,5	-41±4	169,8±11,6	55,4±2,6	mod

General rules of proteins structure organization Depending in the length, size of connections and angles, up to four typical closely packed conformations of the consequent alpha-helixes can be distinguished and itemized.

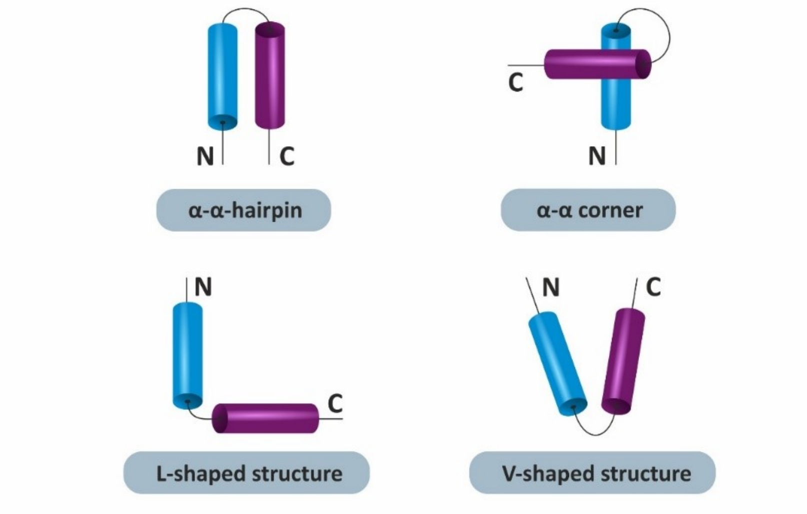

Feature	α-α corner	α-α hairpin	L-shaped structure	V-shaped structure
Folding of alpha helices	orthogonal	antiparallel	dense laterally beveled	dense laterally beveled
Projection and axes of helices	intersect	not intersect	not intersect	not intersect
Inter-planar (**d**) and minimal (**r**) distances	d=r	**d** varied in a wide range	–	–
Distance between turns	9-11 Å	–	–	–
Angle between helices (ϕ)	70-110°	0-30°	90°	50°
Length of linker	4-5 amino acid residues	varies	varies	varies

Supplementary Figure S3: Types and rules for qualification of secondary structures comprised of two distinct α-helices and unstructured
